# Traversing the epigenetic landscape: DNA methylation from retina to brain in development and disease

**DOI:** 10.3389/fncel.2024.1499719

**Published:** 2024-11-29

**Authors:** Chunxiu Xu, Xuefei Fu, Huan Qin, Kai Yao

**Affiliations:** ^1^Institute of Visual Neuroscience and Stem Cell Engineering, Wuhan University of Science and Technology, Wuhan, China; ^2^College of Life Sciences and Health, Wuhan University of Science and Technology, Wuhan, China

**Keywords:** DNA methylation and DNA demethylation, diabetic retinopathy, age-related macular degeneration, glaucoma, schizophrenia, autism spectrum disorder, intellectual disability, Alzheimer’s disease and Huntington’s disease

## Abstract

DNA methylation plays a crucial role in development, aging, degeneration of various tissues and dedifferentiated cells. This review explores the multifaceted impact of DNA methylation on the retina and brain during development and pathological processes. First, we investigate the role of DNA methylation in retinal development, and then focus on retinal diseases, detailing the changes in DNA methylation patterns in diseases such as diabetic retinopathy (DR), age-related macular degeneration (AMD), and glaucoma. Since the retina is considered an extension of the brain, its unique structure allows it to exhibit similar immune response mechanisms to the brain. We further extend our exploration from the retina to the brain, examining the role of DNA methylation in brain development and its associated diseases, such as Alzheimer’s disease (AD) and Huntington’s disease (HD) to better understand the mechanistic links between retinal and brain diseases, and explore the possibility of communication between the visual system and the central nervous system (CNS) from an epigenetic perspective. Additionally, we discuss neurodevelopmental brain diseases, including schizophrenia (SZ), autism spectrum disorder (ASD), and intellectual disability (ID), focus on how DNA methylation affects neuronal development, synaptic plasticity, and cognitive function, providing insights into the molecular mechanisms underlying neurodevelopmental disorders.

## 1 Introduction

DNA methylation has garnered widespread attention due to its crucial role in both physiological and pathological processes, particularly in development (Roost et al., 2017), aging (Unnikrishnan et al., 2019), and neurogenesis (Altuna et al., 2019) in the central nervous system (Holliday and Pugh, 1975). Its involvement in tissue development, particularly in the retina and brain, has been extensively documented (Jabari et al., 2022; Lu et al., 2023).

**Figure 1:**
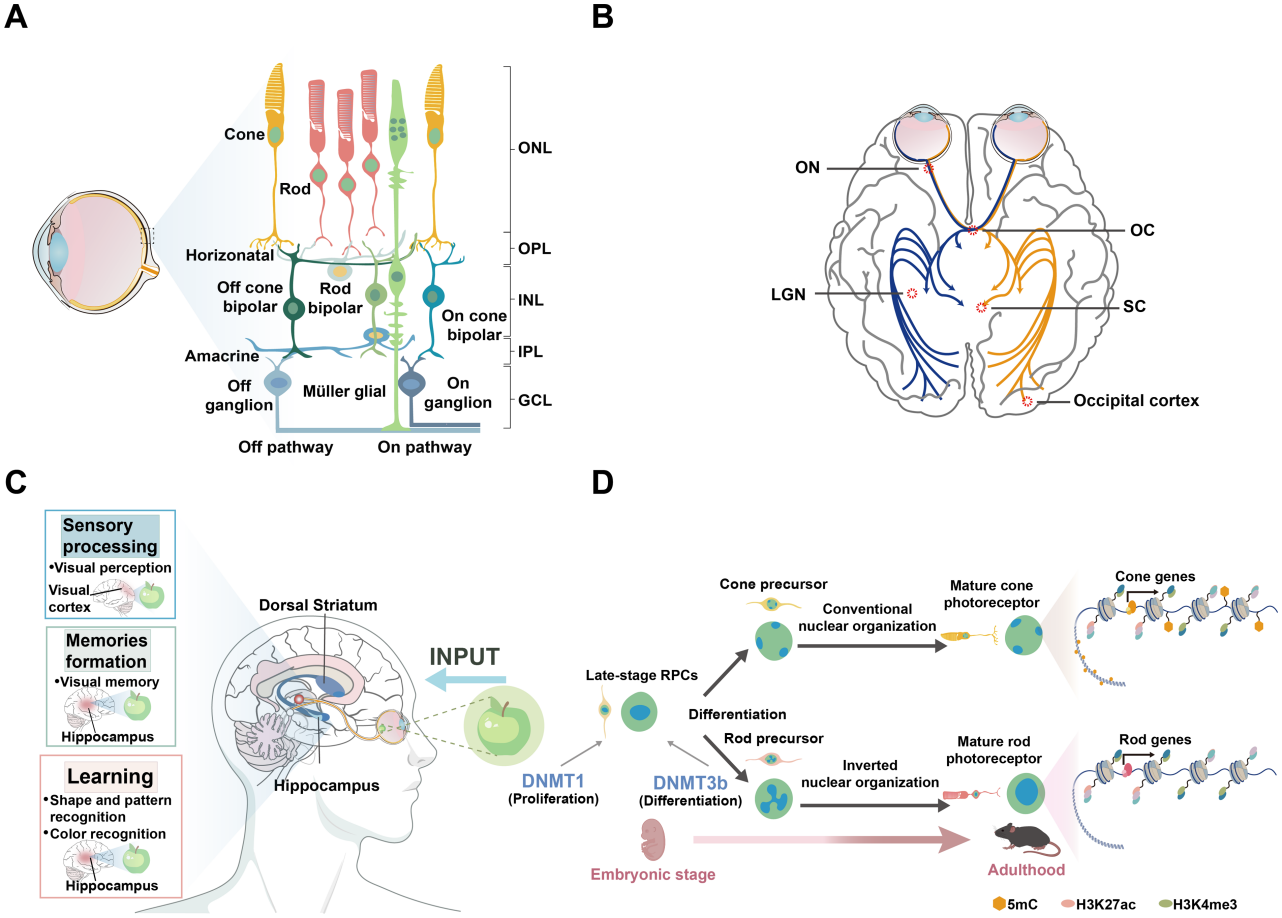
Regions associated with neurodegenerative diseases in the brain and retina. **(A)** Schematic diagram of the human retina, showing the organization of different cell layers. ONL, outer nuclear layer; OPL, outer plexiform layer; INL, inner nuclear layer; IPL, inner plexiform layer; GCL, ganglion cell layer. **(B)** The basic structure of the visual pathway. Axons of retinal ganglion cells (RGCs) form the ON, which extends to the LGN and SC in the brain. ON, optic nerve; OC, Optic chiasm; LGN, lateral geniculate nucleus; SC, superior colliculus. **(C)** The illustration presents the brain structures, including the hippocampus and dorsal striatum, which are associated with Alzheimer’s disease (AD) and Huntington’s disease (HD), respectively. It also highlights the brain functions involving the retina, such as sensory processing, memory formation, and learning. **(D)** The illustration depicts the differentiation of retinal progenitor cells from the embryonic stage to adulthood into various photoreceptors in mice, highlighting the distinct nuclear organization of rod and cone cells. Epigenetic markers such as 5mC, H3K27ac, and H3K4me3 are shown to illustrate chromatin modifications during this process.

DNA methylation is vital for cell growth and differentiation, directly impacting tissue homeostasis (Rajanala and Upadhyay, 2024). In addition to brain development, DNA methylation plays a key role in regulating cell fate during retinal maturation. The retina consists of five major types of neurons and Müller glial cells (MGs) (Zhao et al., 2024; Figure 1A). Retinal development is characterized by the differentiation of neural progenitor cells (NPCs) into various retinal cell types (Figure 1D), a process tightly regulated by DNA methylation, enabling the transition of the retina from embryonic to mature stages (Singh et al., 2017; Rhee et al., 2012). Furthermore, DNA methylation is implicated in retinal diseases such as age-related macular degeneration (AMD), diabetic retinopathy (DR), and glaucoma.

The retina is considered an extension of the CNS, transmitting visual signals to the visual cortex and lateral geniculate nucleus (LGN) via the optic nerve, influencing functions in regions such as the hippocampus (London et al., 2013; Figures 1B,C). The unique physical structure of the eye allows it to exhibit specialized immune responses similar to those in the brain. Several brain diseases, including psychiatric disorders such as schizophrenia (SZ), manifest visual abnormalities or symptoms in the retina (Komatsu et al., 2024). For instance, retinal changes have been observed in patients with schizophrenia, reflecting broader CNS dysfunction. Additionally, in AD, visual abnormalities often appear in the early stages (Donato et al., 2023), with amyloid beta (Aβ) plaque formation and tau protein hyperphosphorylation observed not only in the brain but also in the retina (Zhao et al., 2024). Similarly, AMD is closely linked to Aβ deposition beneath the retinal pigment epithelium (Ding et al., 2011), and glaucomatous degeneration of retinal ganglion cells (RGCs) is associated with p-tau (Liu et al., 2018). Accordingly, this review delves further into the role of DNA methylation in brain-related diseases such as schizophrenia, AD, and HD. To provide a more comprehensive exploration of the role of DNA methylation in neurodevelopmental disorders, we have also expanded the discussion on DNA methylation regulation in autism spectrum disorder (ASD) and intellectual disability (ID).

While DNA methylation has been studied in various tissues, its precise role in mediating interactions between the retina and the brain, and its implications in retinal and brain pathologies remain poorly understood. This review aims to clarify the complex roles of DNA methylation in both retinal and cerebral development, as well as their associated pathologies. We first provide an overview of the critical functions of DNA methylation and demethylation in retinal development and cellular differentiation, followed by an in-depth discussion of their diverse roles in retinal diseases. Given the close connection between the visual system and the CNS, and the fact that ocular symptoms often precede neurological manifestations, we also investigate the involvement of DNA methylation in brain development and diseases. By integrating recent advances, this review highlights the distinct regulatory mechanisms of DNA methylation along the retina-brain axis and offers novel insights into its therapeutic potential for disorders.

### 1.1 DNA methylation and DNA demethylation

**Figure 2:**
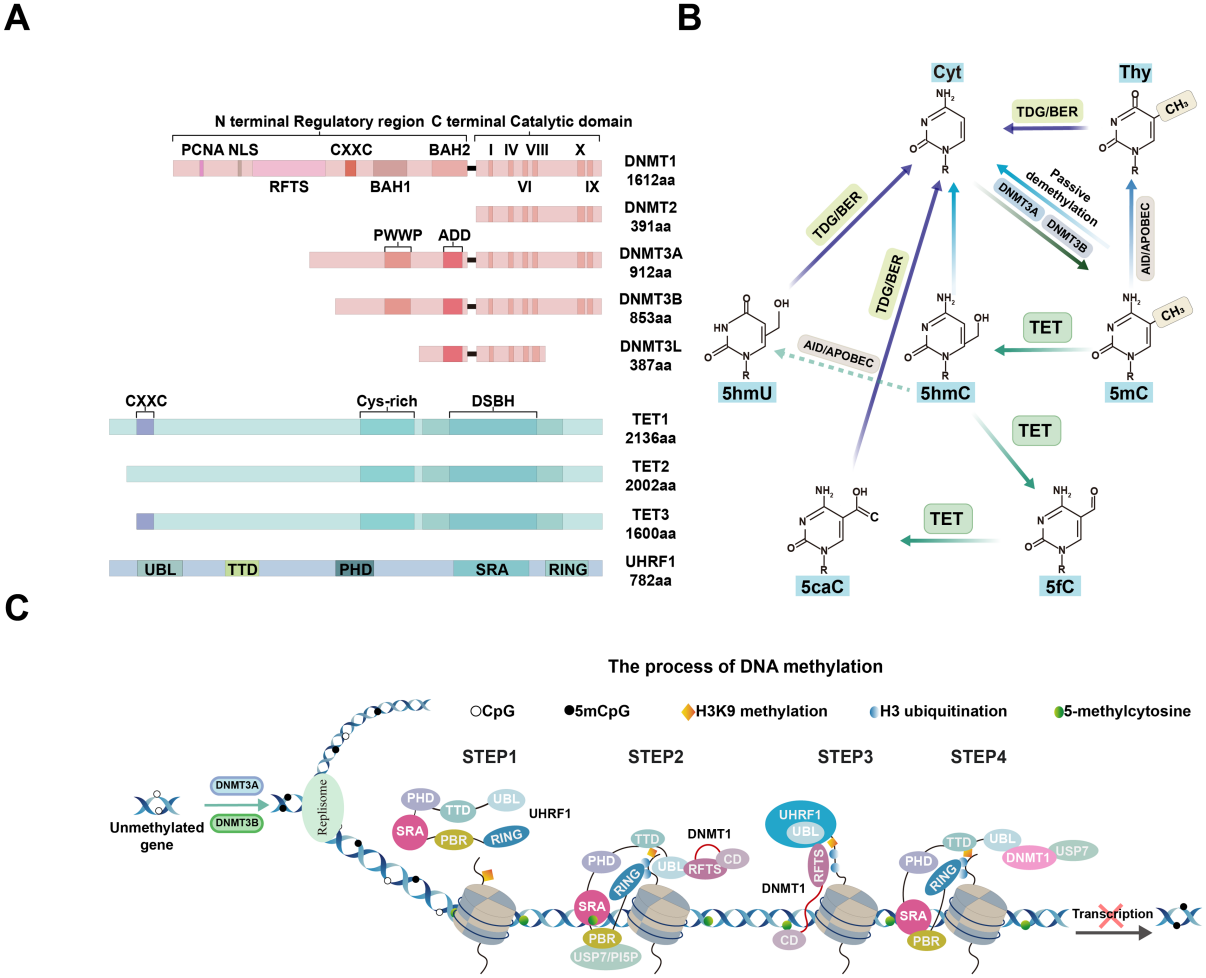
Epigenetic modification factors and processes. **(A)** Schematic representations of the domain structures of human DNMT and TET isoforms, along with mUHRF1, are shown. PCNA, PCNA-interacting domain; NLS, nuclear localization signal; RFTS, replication foci-targeting sequence; CXXC, two cysteines separated by two other residues; BAH1/2, tandem bromo-adjacent homology; PWWP, Pro-Trp-Trp-Pro; ADD: ATRX-DNMT3-DNMT3L. DSBH, double-stranded β-helix; TTD, tandem tudor domain; PHD, plant homeodomain; SRA, Set and Ring Associated domain; RING, Really Interesting New Gene finger domain. **(B)** TET-mediated DNA demethylation pathway diagram. (1) Initial methylation: DNMTs catalyze the formation of 5mC. (2) Oxidation by TET proteins: 5mC is gradually oxidized by TET enzymes to generate 5hmC, 5fC, and 5caC. 5mC: 5-methylcytosine; 5hmC: 5-hydroxymethylcytosine; 5fC: 5-formylcytosine; 5caC: 5-carboxylcytosine. (3) Repair and demethylation: 5hmC, 5fC, and 5caC are excised by thymine DNA glycosylase (TDG) through the base excision repair (BER) pathway, ultimately restoring cytosine and completing the demethylation cycle. Additionally, passive demethylation occurs through dilution during cell division. **(C)** Mechanisms responsible for maintaining DNA methylation by the DNMT1/UHRF1 complex. This section illustrates the role of the DNMT1/UHRF1 complex in recognizing hemimethylated DNA and recruiting necessary enzymes for maintaining methylation patterns during DNA replication. Epigenetic modifications such as H3K9 methylation and H3 ubiquitination are also shown as key regulatory elements in this process.

DNA methylation is a crucial biochemical modification mechanism that involves the precise incorporation of methyl groups into DNA molecules to form 5-methylcytosine (5mC). This process can occur in multiple stages: establishment (*de novo* DNA methylation), maintenance, and demethylation. In mammals, DNA methylation is tightly regulated by the DNMTs family (Figure 2A). DNMT3A and DNMT3B establish new DNA methylation patterns during embryonic development and adulthood (Figure 2C), while DNMT1 maintains these patterns during cell division through a replication mechanism (Goll and Bestor, 2005). Although the primary function of DNMT1 is maintenance methylation, it also participates in *de novo* methylation in specific contexts, such as during oocyte development (Li et al., 2018) and the regulation of retrotransposons (Haggerty et al., 2021).

DNA methylation is most commonly found in CpG dinucleotide regions, which exhibit high stability in CpG islands enriched in promoters. Despite minor variations at certain CpG sites, the overall methylation patterns remain remarkably stable across successive cell divisions. This stability is primarily due to the coordinated actions of DNMT1 and related cofactors, which collectively ensure genomic integrity during development and in response to environmental challenges (Bird, 2002). The flexibility of DNA methylation is evident during biological development, particularly during fertilization and germ cell-specific differentiation, where DNA methylation undergoes comprehensive reprogramming. This suggests that DNA methylation is not static but serves as a highly adaptable tool for epigenetic regulation (Smith and Meissner, 2013). In this regulatory process, DNA methylation can act as a ‘silencing signal’ to repress gene activity in adjacent regions or as an ‘activating catalyst’ to promote transcription within specific gene bodies (Jones, 2012).

This dynamic nature of DNA methylation is particularly evident during human brain evolution. While CpG dinucleotide methylation remains stable in many contexts, other forms of methylation, such as CH methylation, play crucial roles during early brain development (Ma et al., 2017). During human brain evolution, CH methylation is almost absent in the fetal brain, rapidly accumulates after birth, and increases in the gene bodies of neurons in the prefrontal cortex, primarily affecting neuronal subtype differentiation during the early brain development phase (Rizzardi et al., 2019). For instance, the specific CH methylation silencing of inhibitory neuron-specific genes suppresses their expression in the genomes of excitatory neurons, thereby promoting the functional specificity of neuronal subtypes. In contrast, there is a marked reduction in CG methylation in neuronal cells and oligodendrocytes in the prefrontal cortex, especially in cis-regulatory regions, leading to the upregulation of downstream genes (Mendizabal et al., 2019). Moreover, the CG hypomethylation in neurons frequently co-occurs with human neuron-specific histone H3-trimethyl-lysine 4 (H3K4me3) modifications, indicating that CG hypomethylation facilitates an active chromatin landscape in the prefrontal cortex in a cell-type-specific manner. Notably, neuron-specific CG methylation is associated with increased genetic susceptibility to schizophrenia, while the loss of CH hypermethylated genomic regions is linked to schizophrenia-associated DNA polymorphisms (Jeong et al., 2021). Notably, while DNA methylation plays a critical role in human brain development, other species have evolved different epigenetic mechanisms. For instance, while most mammals maintain high levels of genomic methylation (Li and Zhang, 2014), methylation has been lost during evolution in certain species, such as *Drosophila* and *C. elegans*, and *chordate O. dioica* (Zemach and Zilberman, 2010).

In mammalian biological systems, 5mC is a key DNA modification. Despite its inherent stability, 5mC can be reverted to an unmodified state. In primordial germ cells, DNA demethylation occurs through both passive and active mechanisms. Passive demethylation occurs when DNMT1 is absent or inhibited during the DNA replication process (Li and Zhang, 2014). Passive demethylation is primarily achieved by the dilution of methylation marks during cell proliferation, while active demethylation relies on the action of ten-eleven translocation (TET) proteins (Messerschmidt et al., 2014). Active demethylation involves the removal of 5fC by thymine DNA glycosylase, coordinated with the base excision repair pathway (Kohli and Zhang, 2013; Wu and Zhang, 2017). The combination of these two mechanisms ensures proper epigenetic reprogramming during the biological development process.

In recent years, research has primarily focused on TET-mediated active demethylation. The TET enzyme family consists of three members: TET1, TET2, and TET3. Each of these enzymes contains a catalytically active C-terminal domain, which includes a Cys-rich region and a double-stranded *β*-helix (DSBH) region. TET1 and TET3 possess a CXXC domain that can recognize unmethylated CpG sites (Lu et al., 2015). TET family proteins mediate the iterative oxidation of 5mC, producing the key intermediate 5-hydroxymethylcytosine (5hmC) (Kohli and Zhang, 2013; Pastor et al., 2013), which can then be further oxidized to 5-formylcytosine (5fC) and 5-carboxylcytosine (5caC) (Wu and Zhang, 2017). Following the oxidation of 5mC to 5hmC by TET1, repair deaminases convert 5hmC to 5-hydroxyluracil (5hmU). Finally, 5hmU is excised by 5hmU glycosylases and repaired by the base excision repair pathway with unmethylated cytosines (Guo et al., 2011; Figure 2B).

## 2 DNMTs and TETs regulate retinal cell development

DNMTs and demethylases assume particular significance in retinal development and maintenance. Retinal development is a dynamic process regulated by DNA methylation and demethylation during the differentiation of retinal progenitor cells (RPCs) into various retinal cell types (Aldiri et al., 2017); however, the role of these pathways may be less critical during RPCs differentiation into non-photoreceptor retinal phenotypes (Dvoriantchikova et al., 2019). Irregularities in the DNA demethylation process of gene promoters during the differentiation of RPCs into photoreceptors may significantly contribute to the development of retinitis pigmentosa (RP) (Dvoriantchikova et al., 2022).

In the mammalian retina, DNMTs collaborate to maintain the homeostasis of photoreceptors and other retinal neurons (Singh et al., 2017; Nasonkin et al., 2011). DNMT1 and DNMT3 are highly expressed, from embryonic day 10.5 onwards, DNMT1 is predominantly expressed in RPCs, while the high expression of Dnmt3a and Dnmt3b at embryonic day 11.5 (Nasonkin et al., 2011). Detailed studies on the developmental trajectory of mouse NPCs have revealed a dynamic shift from Dnmt3b to Dnmt3a expression, which occurs in parallel with the expression of Dnmt1 in early-born neurons, collectively mapping the complex differentiation landscape of retinal cells (Watanabe et al., 2006). Dnmt1-dependent DNA methylation is essential for the retinal progenitor pool expansion and for the photoreceptor terminal differentiation and retinal neuron survival (Rhee et al., 2012). DNMT3B is implicated in the differentiation of RPCs into precursors of bipolar, horizontal, and photoreceptor cells (Cvekl and Mitton, 2010).

The cone and rod specific genes exhibit cell-specific differential DNA methylation patterns during retinal development and maturation, and are inversely correlated with gene expression. In human and mouse fetal retinas, the promoters of phototransduction and rod cell-specific genes exhibited high methylation levels (Dvoriantchikova et al., 2019; Mo et al., 2016), which decreased as RPCs differentiated into photoreceptors, and are accompanied by increased expression (Solovei et al., 2009). Between postnatal days 6 and 10, rod-specific phototransduction genes such as *Rho*, *Gnat1*, and *Cnga1* are significantly upregulated. Moreover, the transcription factor NRL, crucial for rod cell fate determination, consistently maintains low DNA methylation levels in rod cells, whereas cone-specific genes exhibit high methylation levels (Kim et al., 2016).

DNMTs alter gene expression patterns by influencing chromatin structure in the nucleus. The nuclei of most nocturnal mammalian rod cells exhibit a unique ‘inverted’ architecture, with dense heterochromatin internally and euchromatin at the periphery (Solovei et al., 2009; Smith et al., 2021). This structure forms during the final stages of rod cell differentiation and is associated with changes in chromatin organization (Smith et al., 2021). Additionally, the nuclei of mouse rod cells are exceptionally small, and the increased chromatin condensation in rod cells may reduce the level of DNA methylation by limiting the accessibility of DNMTs. In contrast, the strong nuclear localization of DNMTs in cone cells highlights the active role of DNA methylation in chromatin remodeling (Mo et al., 2016).

Extensive analysis of *Dnmt* gene mutations in the retina further highlights the crucial role of DNA methylation in determining retinal cell fate. In tissue-specific triple mutant mice, the loss of all three Dnmts resulted in the reorganization of photoreceptor structure, severe dysregulation of phototransduction and synaptic function genes, and was accompanied by global hypomethylation (Singh et al., 2017). Notably, the conditional knockout (cKO) of *Dnmt1* using the Chx10-Cre line led to the rapid death of photoreceptors and other neuronal cell types at birth, while the differentiation process of RPCs remains unaffected (Rhee et al., 2012). Conversely, the conditional knockdown of *Dnmt1* during early eye development using the Rx-Cre line did not result in cell fate defects, except in cone cells, despite the complete absence of photoreceptor outer segments, underscoring the indispensable role of Dnmt1-mediated DNA methylation in retinal differentiation (Nasonkin et al., 2013). In zebrafish models, the deletion of *dnmt3* caused severe defects in the brain and retina, severely impairing normal neurogenesis. Interestingly, knockdown of the *Lef1* gene can partially rescue the neurogenesis defects caused by the absence of *dnmt3* (Rai et al., 2010). DNMT1 also maintains the proliferation of retinal stem cells (RSCs), gene expression, and the suppression of endogenous retroelements (REs) in the ciliary marginal zone (CMZ) of the zebrafish retina. Although the function of DNMT2 remains controversial, morpholino-mediated knockdown of *dnmt2* in zebrafish embryos has shown its involvement in the retinal differentiation process (Rai et al., 2007).

DNA demethylation is also involved in the tissue-specific development of the eye, particularly in retinal formation. It is essential for the acquisition of normal retinal neuron phenotypes during retinal development (Lu et al., 2023). In *tet2^−/−^*; *tet3^−/−^* zebrafish mutants, the majority of RGCs failed to complete terminal differentiation and axon formation, while few photoreceptors were unable to form outer segments or fully differentiate into mature neurons (Seritrakul and Gross, 2017). Furthermore, the *Wnt* and *Notch* signaling pathways act downstream of tet2 and tet3 activity to regulate the differentiation and morphogenesis of RGCs (Seritrakul and Gross, 2017). Table 1 summarizes the phenotypic changes observed in mice lacking DNA methylation regulators and the associated human diseases.

**Table 1:** Phenotypes associated with the loss of epigenome modifier genes in mice.

Gene	Function	Type	Target	Phenotypes of gene mutant mice	Human diseases	Refs
DNMT1	Maintenance of DNA methylation	Rx-Cre	RPC	Defects in photoreceptor outer segment biogenesis Impaired retinal pigment epithelium morphology	Hereditary sensory and autonomic neuropathy type 1E, (HSAN1E; OMIM#614116) Autosomal dominant cerebellar ataxia, deafness, and narcolepsy (ADCA-DN; OMIM#604121)	Singh et al. (2017)
		Chx10-Cre	RPC	Abnormal differentiation of photoreceptors Defective cell cycle progression		Rhee et al. (2012)
DNMT3A	*De novo* DNA methylation	Rx-Cre	RPC	Diminished outer plexiform layer Absence of outer segments Disorganization of synaptic terminals Global hypomethylation	Tatton-Brown-Rahman syndrome (TBRS; OMIM#615879) Leukemia, acute myelogenous (AML; OMIM#601626) Heyn-Sproul-Jackson syndrome (HESJAS; OMIM#618724)	Singh et al. (2017)
DNMT3B	*De novo* DNA methylation	Rx-Cre	RPC		Immunodeficiency, centromeric instability and facial anomalies syndrome 1 (ICF1; OMIM#242860) Facioscapulohumeral muscular dystrophy 4 (FSHD4; OMIM#619478)	
DNMT3C	*De novo* DNA methylation cofactor	Germline	ESC	Males were sterile Defect in transposon silencing during spermatogenesis	Mouse-specific gene, not directly associated with human diseases	Barau et al. (2016)
DNMT3L	DNMT3A/3B cofactor	Germline	ESC	Unable to establish imprinting in females Fail to establish methylation imprints in the oocytes Male sterility	No direct association with human diseases, involved in epigenetic regulation in germ cells.	Hata et al. (2002)
UHRF1	DNMT1 cofactor	Germline	ESC	Massive global losses of DNA methylation Embryonic lethality		Felle et al. (2011)
TET1	DNA demethylation	Germline	ESC	Developmental defects in chimeric embryos Females had smaller ovaries and reduced fertility		Dawlaty et al. (2013)
TET2	DNA demethylation	Germline	ESC	Increased hematopoietic stem cell self-renewal	Polycythemia vera (PV; OMIM#263300); Myelodysplastic syndrome (MDS; OMIM#614286) Immunodeficiency-75 with lymphoproliferation (IMD75; OMIM#619126)	Gu et al. (2011)
TET3	DNA demethylation	Germline	ESC	Female mice with Tet3 depletion in the germ line show reduced fecundity Paternal genome underwent active DNA demethylation		

DNMT, DNA methyltransferase; RPC, retinal progenitor cell; ESC, embryonic stem cell.

DNA methylation changes are present in dying retinal neurons. Increased cytosine methylation has been observed in dying photoreceptors in the rd1, rd2, P23H, and S334ter RP models. In rd1 mice, the severe chromatin structure changes that occur during retinal degeneration are linked to the overexpression of Dnmt3a (Farinelli et al., 2014). Furthermore, elevated levels of 5mC and 5hmC in the rd1 mouse retina are associated with both classical caspase-dependent apoptosis and caspase-3-independent cell death (Wahlin et al., 2013). Treatment with the demethylating agent decitabine attenuated photoreceptor death in organotypic explants, highlighting the therapeutic potential of targeting DNA methylation in retinal diseases (Farinelli et al., 2014). Thus, DNA methylation is involved not only in the differentiation and tissue-specific development of the retina, but also plays a crucial role in regulating gene expression throughout retinal development and aging.

## 3 DNA methylation in eye diseases

As age advances, the retina undergoes significant changes, including decreased retinal thickness and impaired visual function (Cavallotti et al., 2004). Underlying this phenomenon, retinal diseases such as AMD, DR and glaucoma often arise, influenced by both genetic and environmental factors. DNA methylation serves as a key epigenetic mark, linking genetic variations and environmental influences, and mediating genetic effects on retinal diseases through gene expression and metabolic pathways (Advani et al., 2023). The aging process is characterized by epigenetic changes, including global heterochromatin formation, nucleosome remodeling and loss, changes in histone marks and changes in DNA methylation status (Kane and Sinclair, 2019). However, research on how aging and its hallmark events, DNA methylation, specifically affect these ocular diseases remains limited (Tsantilas et al., 2021; Otteson, 2011). So far, there have been no studies on epigenomic changes during the normal aging process of the human retina. Therefore, we explored the alterations in DNA methylation patterns and gene expression in retinal diseases to elucidate their roles in disease progression and their potential as therapeutic targets.

### 3.1 Diabetic retinopathy

**Figure 3:**
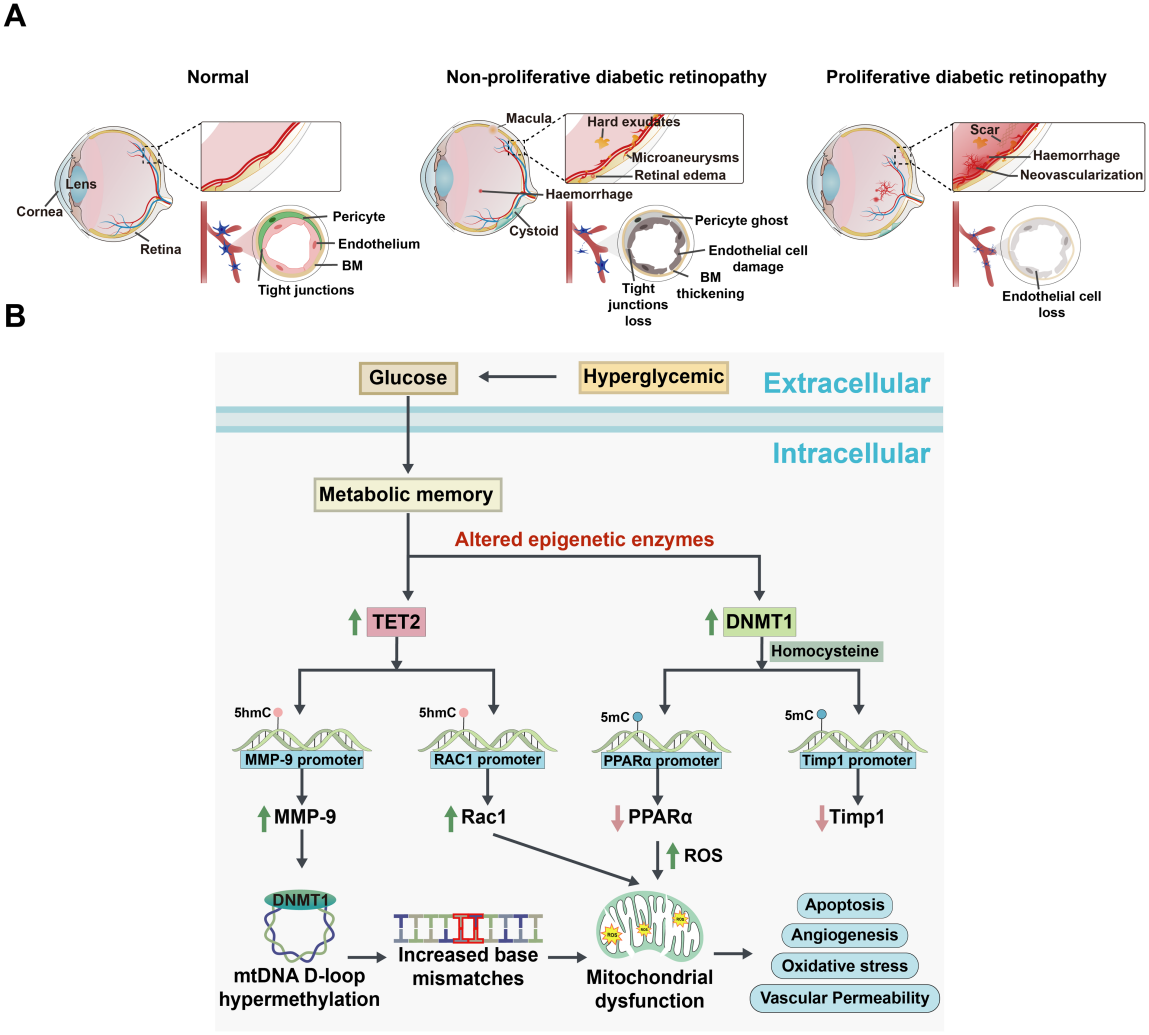
Comparison of changes in the eye among normal, non-proliferative diabetic retinopathy (NPDR), and proliferative diabetic retinopathy (PDR), and the signaling pathways of DNA methylation alterations associated with DR development. **(A)** Schematic diagram illustrating changes in the eye under normal, NPDR, and PDR conditions. Normal eye: The retinal vascular network is well-organized and intact, with no signs of abnormal proliferation or damage. The vascular barrier is fully functional. NPDR eye: Shows microvascular abnormalities, including tortuosity, dilation, and microaneurysms. The vascular barrier is compromised, potentially accompanied by mild edema or exudates. PDR eye: Displays significant retinal damage with severe compromise of the vascular barrier, resulting in extensive exudation and hemorrhage. Notable features include extensive neovascularization. BM: basement membrane. **(B)** Mechanisms of DNA methylation changes and cellular function alterations in a hyperglycemic environment. High Glucose induces oxidative stress and epigenetically modulates the expression of TET2 and DNMT1. Elevated TET2 levels decrease methylation of the matrix metalloproteinase-9 (*MMP-9*) and Ras-related C3 botulinum toxin substrate 1 (*Rac1*) promoters, enhancing their expression and disrupting mitochondrial homeostasis and signaling pathways. Concurrently, increased DNMT1 activity leads to hypermethylation of the Timp1 and proliferator-activated receptor alpha (*PPARα*) promoters, suppressing their expression. These changes result in increased MMP-9 activity, compromised antioxidant defenses, and elevated apoptosis. Additionally, high Glucose levels correlate with increased methylation in the mitochondrial D-loop and base mismatches, impairing mitochondrial biogenesis and function, thereby affecting cellular energy metabolism and survival.

Diabetic retinopathy is one of the leading causes of moderate to severe vision loss worldwide, categorized into early non-proliferative and late proliferative stages (Figure 3A; Jampol et al., 2020). According to the latest World Health Organization report, approximately 537 million people globally are affected by diabetes, with about one-third suffering from DR (Hossain et al., 2024). In the retina subjected to diabetic or high-glucose conditions, dysregulated expression of modifying enzymes impacts DNA and histone regulation, resulting in alterations to the epigenetic modifications of key genes essential for maintaining mitochondrial homeostasis. This process differentially influences the development and progression of DR (Kowluru et al., 2015; Kowluru and Mishra, 2015). Notably, the levels of DNMTs and TETs are abnormal in the retina and its vascular system in diabetic rats, suggesting that DNA methylation may play a role in the pathogenesis of DR (Kowluru et al., 2016; Liu and Cui, 2021).

We further investigated the role of aberrant DNA methylation in driving the pathogenic mechanisms of oxidative stress (Duraisamy et al., 2018), inflammation (Chen et al., 2019), and neovascularization (Berdasco et al., 2017) in DR. Additionally, a noteworthy phenomenon in DR is that even with tight glycemic control, persistent DNA methylation-hydroxymethylation activity contributes to ‘metabolic memory’ (Kowluru et al., 2016; Mishra and Kowluru, 2016), which is a significant driver of DR progression. Therefore, targeting these epigenetic mechanisms may offer new therapeutic opportunities to disrupt the vicious cycle of metabolic memory.

The retina is particularly vulnerable to oxidative damage, diabetes-induced increases in reactive oxygen species (ROS) can harm mitochondria and accelerate capillary cell apoptosis, ultimately leading to DR (Pickering et al., 2018; Robles-Rivera et al., 2020). Oxidative stress influences DNA methylation by activating DNMTs or TETs, and this methylation, in turn, exacerbates oxidative stress (Afanas'ev, 2014; Ziech et al., 2011). The abnormal DNA methylation mechanisms in related genes, such as polymerase gamma (*POLG*), Matrix metalloproteinase-9 (*MMP-9*), Peroxisome Proliferator-activated Receptor *α* (*PPARα*), and Ras related C3 botulinum toxin substrate 1 (*Rac1*), elucidated this process.

Notably, DNMT1 is the only DNMT enzyme that is activated and upregulated in DR (Kowluru et al., 2016; Mishra and Kowluru, 2016). *POLG* is crucial for mtDNA replication by binding to the D-loop region and plays a key role in repairing mitochondrial DNA (mtDNA) damage (Facchinello et al., 2021). However, the persistent activation of DNMT1 led to hypermethylation of the *POLG1* promoter in the retinal endothelial cells (RECs) that were exposed to normal glucose following exposure to high glucose. This hypermethylation impaired *POLG*’s binding affinity to mtDNA, reduced its transcriptional activity and accelerated ROS production. Similar results were observed in the streptozotocin-diabetic rats after 3 months of good glycemic control that had followed 3 months of poor glycemic control (Tewari et al., 2012). The resulting accumulation of superoxide radicals further exacerbates oxidative stress, forming a vicious cycle that accelerates the progression of DR.

*MMP-9* contributes to mitochondrial damage and is regulated by DNA methylation (Mishra and Kowluru, 2017). Under diabetic conditions, elevated homocysteine levels promoted the upregulation of DNMTs and TET enzymes in the retina, resulting in increased 5mC levels at the *Timp1* promoter and elevated 5hmC levels at the *MMP-9* promoter. This change not only suppressed *Timp1* transcription but also activated *MMP-9*, leading to mitochondrial damage and exacerbated oxidative stress (Mohammad and Kowluru, 2020; Figure 3B). Additionally, in RECs, hyperglycemia further enhanced the binding of DNMT1 and TET2, amplifying *MMP-9* activation and worsening mitochondrial dysfunction. Studies have shown that the manganese superoxide dismutase (MnSOD) mimetic MnTBAP effectively prevented mitochondrial damage by reducing DNMT1 binding to the *MMP-9* promoter (Kowluru and Shan, 2017), suggesting that targeting DNA methylation to inhibit *MMP-9* could suppress oxidative stress and serve as a promising therapeutic strategy for diabetic retinopathy.

*PPARα*, a transcription factor critical for oxidative stress, was downregulated under high-glucose conditions in human retinal capillary pericytes (HRCPs) due to elevated levels of DNMT1, which promoted hypermethylation of the *PPARα* promoter. This downregulation contributed to increased apoptosis and elevated production of ROS (Ramakrishnan et al., 2016). Furthermore, treatment with decitabine effectively inhibited *PPARα* promoter methylation and reduce the destruction of retinal cells (Zhu et al., 2021). These findings provide strong evidence that DNMT1-mediated DNA methylation of *PPARα* accelerates apoptosis and ROS production.

Finally, *Rac1*, a key component of NADPH oxidase (Nox), is necessary for NOX2 activation and ROS generation (Kowluru et al., 2014), became both functionally and transcriptionally active in diabetes due to the co-activation of DNMTs and TETs. DNMTs increased 5mC levels, which were subsequently converted to 5hmC by TET enzymes, thereby facilitating *Rac1* transcription. The upregulation of Rac1 expression contributed to mitochondrial damage and accelerated capillary cell apoptosis. Notably, when the TET enzyme was knocked down, NF-κB binding to the *Rac1* promoter and *Rac1* expression were suppressed, effectively preventing apoptosis in capillary cells (Duraisamy et al., 2018). These results highlight the potential of targeting DNA methylation pathways as a therapeutic strategy for DR. However, future studies should explore whether inhibiting these pathways could introduce off-target effects or interfere with other essential cellular processes.

Oxidative stress regulates inflammatory signaling pathways by selectively oxidizing NF-κB through ROS, thereby modulating the expression of inflammatory factors (Warnatsch et al., 2017). In DR, inflammation enhances retinal angiogenesis and microvascular damage through the activation of pro-inflammatory cytokines (Forrester et al., 2020). One of the key regulators of oxidative stress in diabetes is thioredoxin-interacting protein (TXNIP), which acts as an endogenous inhibitor of the antioxidant thioredoxin (TRX). TXNIP has been implicated in the processes associated with diabetes and its vascular complications (Perrone et al., 2009). DNA hypomethylation of *TXNIP* has been shown to promote the elevation of inflammatory biomarkers, including *VCAM-1*, *ICAM-1*, *MMP-2*, *sRAGE*, and *P-selectin* (Xiang et al., 2021), while chronic increases in these biomarkers can impaire the signaling pathways necessary for pericyte survival (Dharmarajan et al., 2023). Conversely, DNMT1-mediated DNA methylation reduced the expression of miR-20a, leading to increased *TXNIP* levels, and ultimately resulted in pyroptosis in RPE cells. Furthermore, hypomethylation of the *TGFB1*, *CCL2* and *TNFSF2* genes was associated with an increased risk of DR (Chen et al., 2020), underscoring the broader role of aberrant DNA methylation in regulating inflammation and cell survival. This suggests the critical importance of DNA methylation in controlling the expression of inflammation-related genes, which contributes to the progression of DR.

As DR progresses, retinal ischemia leads to the upregulation of proangiogenic factors, which include pathological neovascularization and accelerating the development of PDR (Nentwich and Ulbig, 2015). Hypomethylated CpG sites in the promoter region of the *ROBP4* gene have been linked to increased *ROBP4* expression under hyperglycemic conditions (Zhao et al., 2023). This finding suggests that DNA methylation plays a role in regulating vascular integrity and neovascularization in DR. Furthermore, *Tet2*, a key regulator of epigenetic modifications, is highly expressed in the retinas of DR models. Elevated levels of *Tet2* are associated with reduced methylation at the *ROBP4* promoter, which led to decreased expression of tight junction proteins such as *ZO1* and occludin, ultimately contributing to neovascularization and exacerbating retinal vascular dysfunction (Zhao et al., 2023; Mohammad et al., 2019; Duraisamy et al., 2019). Additionally, extracellular matrix protein 1 (*ECM1*), a gene critical for angiogenesis, is also regulated by *Tet2*. Increased *Tet2* expression resulted in hypomethylation of the *ECM1* promoter, thereby enhancing its transcription in RECs. Higher *ECM1* levels were implicated in promoting retinal neovascularization and fibrovascular membrane proliferation, further driving the progression of PDR (Cai et al., 2024). Collectively, these studies demonstrate the critical role of DNA methylation in regulating oxidative stress, mitochondrial dysfunction, cell apoptosis, and neovascularization in the progression of DR.

### 3.2 Age-related macular degeneration

**Figure 4:**
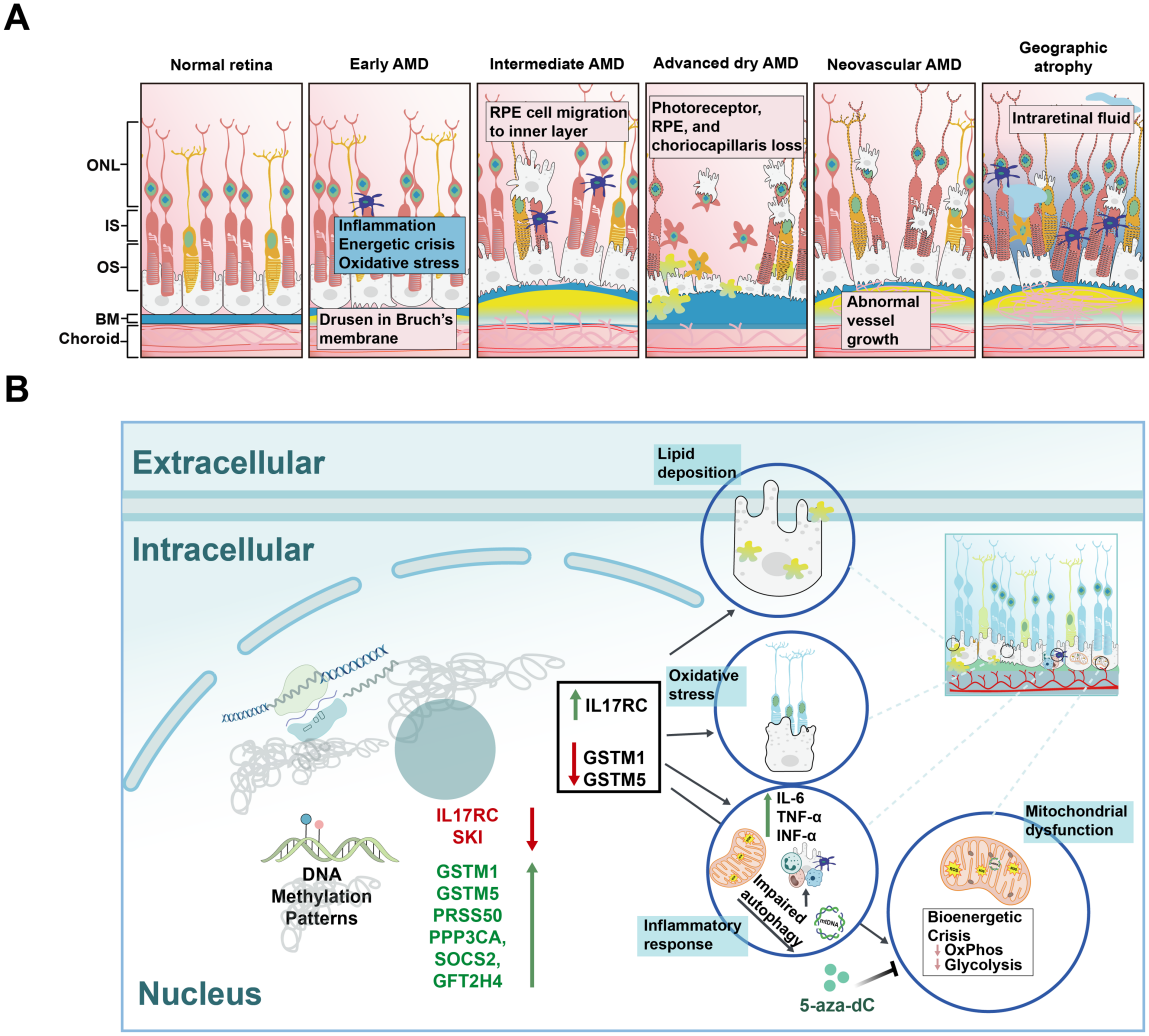
Schematic diagrams of normal retina, different types of age-related macular degeneration (AMD), and risk factors for AMD. **(A)** Schematic diagrams illustrating the normal retina and various stages of AMD. Normal Retina: Displays a healthy retinal structure with distinct layers, including the photoreceptor cells, without any pathological signs. Early AMD: Characterized by the appearance of small yellow deposits called drusen on BM, indicating early retinal changes. Intermediate AMD: Shows increased drusen size and number, along with pigmentary changes in the RPE, indicating progression of retinal degeneration. Advanced dry AMD: Marked by extensive drusen accumulation, significant RPE and photoreceptor cell loss, and widespread retinal atrophy. Neovascular AMD: Characterized by abnormal blood vessel growth (neovascularization), leading to hemorrhage, fluid leakage, and severe retinal damage. Geographic atrophy: Further characterized by the progressive enlargement of atrophic areas in the retina, leading to substantial vision loss. IS: inner segments; OS: outer segments; BM: Bruch’s membrane. **(B)** The impact of DNA methylation on AMD mechanisms. Hypomethylation of *IL17RC* leads to its upregulation, while hypermethylation of *GSTM1* and *GSTM5*, along with their downregulation, promotes a series of biological processes in RPE cells, including lipid deposition, oxidative stress, inflammatory response, and mitochondrial dysfunction.

AMD is a leading cause of irreversible central vision loss (Guymer and Campbell, 2023). Early stages of AMD are characterized by the accumulation of lipoproteinaceous debris between the photoreceptors and the retinal pigment epithelium (RPE) or beneath the RPE cells. As the disease progresses, central choroidal neovascularization develops, ultimately leading to the formation of a disciform scar, that significantly impairs visual function (Guillonneau et al., 2017; Figure 4A). In a study, the twin with a higher dietary intake of vitamin D, betaine, or methionine exhibited earlier stages of AMD, suggesting that epigenetic modifications may play a crucial role in the pathogenesis of the disease (Seddon et al., 2011). Additionally, in AMD models, the ectopic expression of DNMT has been shown to influence the regulation of genes involved in angiogenesis, inflammation, and oxidative stress, indicating that DNA methylation is a significant factor in the progression of AMD (Maugeri et al., 2018; Huang et al., 2018).

The overexpression of vascular endothelial growth factor (*VEGF*) in the RPE and choroid facilitates the penetration of CNV through Bruch’s membrane into the subretinal space, thereby promoting the progression of AMD (Thomas et al., 2021). mtDNA variants have been shown to regulate methylation profiles and the transcription of genes related to inflammation and angiogenesis, particularly the expression of *VEGF*. Human RPE cybrid cell lines were developed by fusing mitochondria-deficient ARPE-19 cells with platelets derived from AMD patients. In these AMD cybrids, the levels of the DNMT3A and DNMT3B genes and proteins were elevated, while DNMT1 expression was reduced. Notably, treatment with the demethylating agent 5-aza-dC resulted in decreased *VEGF* expression, suggesting a link between methylation and angiogenesis (Nashine et al., 2019). Additionally, the mtDNA in H and J cybrids is derived from the H or J haplogroup, respectively, with the H haplogroup being protective against AMD and the J haplogroup associated with an increased risk. Following treatment with 5-aza-dC, the expression levels of nuclear genes (*CFH*, *EFEMP1*, *VEGFA*, and *NFkB2*) in both H and J cybrids became comparable, indicating that mtDNA variants may modulate AMD progression through their effects on DNA methylation.

In addition to the mechanisms of angiogenesis, dysregulated pro-inflammatory cytokine signaling represents another critical factor in the pathogenesis of AMD. In AMD patients, elevated levels of complement component 5a (C5a) amplify inflammatory responses by increasing the expression of pro-inflammatory cytokines such as interleukin (IL)-22 and IL-17 (Liu et al., 2011). These cytokines promote the hypomethylation of the *IL17RC* gene, leading to its increased expression and subsequent elevation of inflammatory factors in retinal (Wei et al., 2012). It is plausible to investigate DNA methylation changes in immune-related genes, as the majority of cell populations consist of immune cells. Furthermore, the dysregulation of the complement system has long been associated with AMD pathogenesis, likely contributing to the chronic inflammation and angiogenesis that drive disease progression.

Oxidative stress also plays a critical role in AMD pathology. Glu*tathione S-transferase isoforms mu1* and *mu5* (*GSTM1* and *GSTM5*), which are vital for protecting cells from oxidative damage, have been found to be hypermethylated in AMD samples compared to age matched controls (Figure 4B; Hunter et al., 2012). A study analyzing data from the RPE/choroid and neurosensory retina of AMD patients, revealed that hypermethylation of the *GSTM1* and *GSTM5* promoters compromised the antioxidant defense of retinal cells. Additionally, *LINE-1* methylation, a surrogate marker of global methylation (Peng et al., 2011), was found to be increased in AMD patients (Maugeri et al., 2019). Sirtuin 1 (*SIRT1*) is involved in regulating inflammation and oxidative stress (Kang et al., 2017). Resveratrol, by modulating *SIRT1* activity, altered DNMT1 and LINE-1 functions in ARPE-19 cells, thereby protecting these cells from apoptosis (Maugeri et al., 2018).

DNA methylation may serve as a promising biomarker for predicting the risk of AMD. Increasing evidence indicates that differentially methylated genes are present in AMD patients compared to their matched controls. For instance, *SMAD2* and *NGFR* have been identified as potential markers for AMD treatment (Wang et al., 2021). A genome-wide methylation analysis of AMD patients identified four hypermethylated genes (*CKB*, *PPP3CA*, *TGFβ*1, and *SOCS2*), which overlap with AMD risk genes in the retinal and choroidal samples (Shen et al., 2020). Additionally, differential gene expression patterns were observed in human retinal cell cybrids containing mitochondria from AMD patients compared to those from age-matched normal subjects (Nashine et al., 2019). Moreover, differential DNA methylation in the protease serine 50 (*PRSS50*) gene in the blood of neovascular AMD patients may help identify novel gene sites contributing to AMD development (Oliver et al., 2015). Thus, DNA methylation may act as a critical mediator, integrating environmental signals such as inflammation and oxidative stress, and ultimately influencing the gene expression pathways involved in AMD pathogenesis (Hunter et al., 2012).

### 3.3 Glaucoma

**Figure 5:**
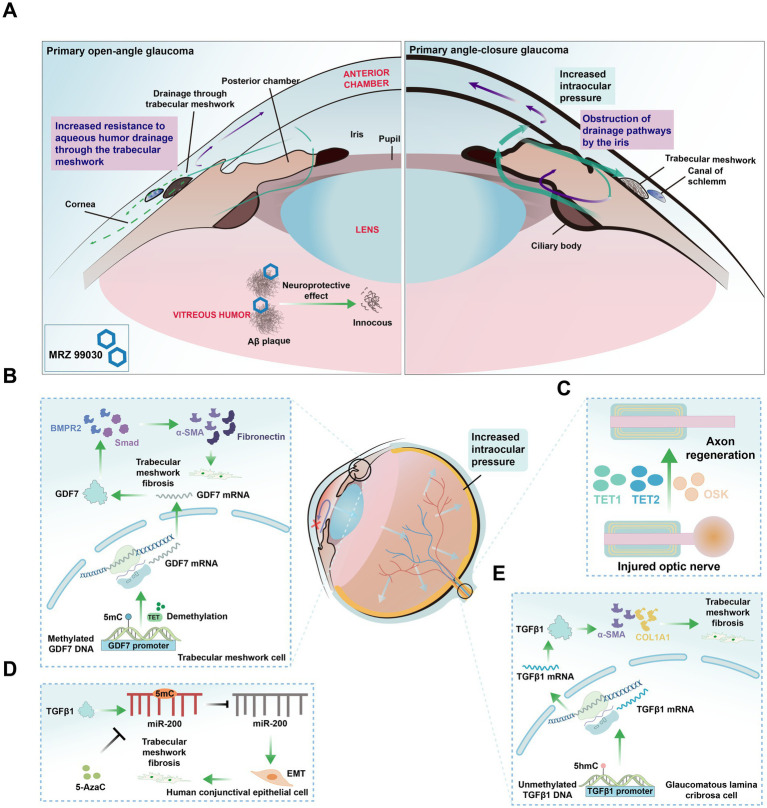
Glaucoma classification and cell type changes related to DNA Methylation alterations. **(A)** Diagram of two types of glaucoma. Primary open-angle glaucoma (POAG): The most common type of glaucoma, characterized by increased resistance to aqueous humor drainage through the trabecular meshwork, leading to elevated intraocular pressure and progressive optic nerve damage. Primary angle-closure glaucoma (PACG): A less common type, characterized by a sudden increase in intraocular pressure due to the obstruction of drainage pathways by the iris, potentially causing acute vision loss. MRZ-99030: A compound that mitigates vision loss in glaucoma by targeting and neutralizing amyloid-beta (Aβ) oligomers. **(B)** Role of TET enzymes in TM fibrosis. TET enzymes maintain hypomethylation of the growth differentiation factor 7 (*GDF7*) promoter, activating the bone morphogenetic protein receptor type 2 (BMPR2) /Smad signaling pathway and inducing the expression of pro-fibrotic genes such as α-smooth muscle actin (*α-SMA*), fibronectin, leading to TM fibrosis and obstruction of aqueous humor outflow. **(C)** Axon regeneration in injured optic nerve. The ectopic expression of *Oct4*, *Sox2*, and *Klf4*, along with TET1 and TET2, restores youthful DNA methylation in RGCs and promotes regeneration of damaged axons. **(D)** *TGFβ1*-induced epithelial-mesenchymal transition (EMT) and DNA methylation changes. *TGFβ1* induces EMT in human conjunctival epithelial cells, leading to increased methylation of the miR-200 loci, which can be reversed by 5-Azacytidine (5-AzaC) treatment. **(E)** DNA methylation alterations in glaucomatous lamina cribrosa cells result in reduced methylation of the *TGFβ1* promoter. This leads to increased expression of TGFβ1, αSMA, and collagen 1α1 (COL1A1), ultimately promoting fibrosis.

Glaucoma, the second leading cause of irreversible blindness worldwide, is characterized by compromised axonal function in the optic nerve head. This impairment initiates a cascade of pathological events, including extracellular matrix remodeling, elevated intraocular pressure, aqueous humor outflow obstruction and the apoptosis of RGCs, ultimately resulting in vision loss (Pitha et al., 2024). By 2040, the global prevalence of glaucoma is expected to rise to 111.8 million (Tham et al., 2014). Although multiple risk factors exist, intraocular pressure (IOP) remains the only therapeutic target; however, it does not fundamentally alter the progression of the disease (Jayaram et al., 2023; Figure 5A). Since DNA methylation is a dynamic and reversible modification process, it has gradually become an important focus for studying the mechanisms of glaucoma, with researchers exploring its role in the onset and progression of the disease.

Recent studies have highlighted the crucial role of aberrant DNA methylation in the pathogenesis of glaucoma (Cai et al., 2020; Greene et al., 2020; Chansangpetch et al., 2018). For instance, hypermethylation of CpG islands in the *HSP70* gene exonic region has been observed in patients with pseudoexfoliation syndrome (PEXS), accompanied by deduced *HSP70* expression and increased DNMT3A activity, suggesting a role for *de novo* methylation in PEXS. As a molecular chaperone, *HSP70* is vital for protein folding and cellular stress response. Its downregulation may impair cellular stress management, promoting protein aggregation and cellular dysfunction, which contribute to PEXS pathogenesis (Hayat et al., 2020).

TNFAIP3, an inhibitor of NF-κB activation and TNF-*α* signaling, was found to exhibit variable expression in glaucoma, which is associated with promoter methylation. Specifically, in glaucomatous samples, *TNFAIP3* expression varied significantly among individuals, with some samples showing increased expression. However, in samples with high promoter methylation, *TNFAIP3* expression was downregulated, which may weaken the inhibition of NF-κB and TNF-α pathways, leading to enhanced inflammation (Yang et al., 2011). This heightened inflammation response can promote RGC apoptosis and optic nerve damage, contributing to glaucoma progression (Baudouin et al., 2021). Furthermore, DNA methylation studies identified DMRs in glaucoma-related genes, such as *PLEKHA7* and *PITX2*, between primary open-angle glaucoma (POAG) patients and normal Schlemm’s canal cells (Fraga and Esteller, 2007), reinforcing the link between methylation and glaucoma pathology.

Aqueous humor outflow obstruction has long been recognized as a central pathological factor in glaucoma. Growth differentiation factor 7 (*GDF7*), a member of the *TGFβ* superfamily, is involved in TM fibrosis. In primary open-angle glaucoma, the TET enzyme maintained the hypomethylation of the *GDF7* promoter in trabecular meshwork (TM) cells (Wan et al., 2021), thereby activating the bone morphogenetic protein receptor type 2 and Smad signaling pathways. This activation induced the expression of pro-fibrotic genes such as *α*-smooth muscle actin and fibronectin, leading to TM fibrosis and obstructed aqueous humor outflow (Wan et al., 2021; Figure 5B). Additionally, DNA methylation affected the expression of glaucoma-related genes, such as *TBX3*, *TNXB1*, *DAXX*, and *PITX2*, all of which are linked to outflow resistance, as identified through genome-wide DNA methylation profiling in cultured human Schlemm’s canal cells (Cai et al., 2020).

Elevated levels of TGFβ1 contribute to the deposition of extracellular matrix (ECM) in the TM and the juxtacanalicular region of Schlemm’s canal (Suri et al., 2018). In glaucomatous lamina cribrosa cells, increased levels of unmethylated DNA in the *TGFβ1* promoter were observed, along with increased expression of *TGFβ*1, *TGFβ2*, *COL1A1*, and *α-SMA* (McDonnell et al., 2016), ultimately promoting fibrosis (Figure 5E). Additionally, *TGFβ1*-induced EMT in human conjunctival epithelial cells was associated with increased miR-200 loci methylation, a change that can be reversed by 5-Azacytidine (5-AzaC) treatment (Rajić et al., 2020; Figure 5D). In trabeculectomy samples, hypomethylation of Alu elements was noted across all glaucoma subtypes, while hypermethylation of *HERV-K* was specific to primary open-angle glaucoma patients, both alterations are associated with TM fibrosis and contribute to increased IOP (Chansangpetch et al., 2018).

Experimental models have demonstrated the potential of inhibiting DNA methylation to reverse glaucoma progression. In mouse models of glaucoma and aged mice, the ectopic expression of the genes *Oct4* (also known as *Pou5f1*), *Sox2*, and *Klf4*, alongside the involvement of TET1 and TET2, has been shown to restore a youthful DNA methylation pattern in RGCs, leading to vision recovery (Lu et al., 2020; Figure 5C). In tenon fibroblasts from patients with pseudoexfoliation glaucoma, increased methylation of the *Lysyl oxidase like 1* promoter was observed, and treatment with 5-AzaC successfully restored its normal expression (Greene et al., 2020). This suggests that DNMTs and TETs could serve as potential therapeutic targets for glaucoma. A comprehensive understanding of the role of methylation in the pathogenesis of glaucoma could facilitate the development of novel treatment strategies that extend beyond merely managing IOP.

## 4 DNA methylation in brain development

CpG methylation is the predominant DNA methylation form in the brain, playing a role in processes such as X-chromosome inactivation. Recent studies have also highlighted non-CG methylation (mCA), especially conserved in the human brain and established by DNMT3A during neuronal maturation (Guo et al., 2014). During early brain development, DNMT3A binds transcriptional regions of lowly expressed genes, promoting the deposition of mCA (non-CpG methylation). This process primarily occurs during the differentiation of neuronal progenitor cells into specific neuronal subtypes, ensuring the correct establishment of neuronal identity and function through the regulation of subtype-specific gene expression. Additionally, DNMT3A works in conjunction with MECP2 to suppress the transcription of lowly expressed genes, and this suppression continues after neuronal differentiation to maintain cellular functional stability. During the differentiation stage, DNMT3A not only facilitates the specialization of neuronal identity but also regulates synapse formation and early neural network development (Li et al., 2022). Mutations in DNMT3A are closely associated with neurodevelopmental disorders, such as autism spectrum disorder and intellectual disabilities, further highlighting its essential role in maintaining neural health during differentiation (Stroud et al., 2017). Changes in methylation levels at key sites lead to adjustments in gene expression, influencing processes such as brain development and disease susceptibility (Miller and Sweatt, 2007; Chiavellini et al., 2022), synaptic plasticity (Muñoz et al., 2016; Zocher et al., 2021), and cell-type differentiation (Wood, 2014). These processes are closely related to cognitive and neurodevelopmental programs, as well as neuropsychiatric disorders that contribute to human uniqueness (Jeong et al., 2021). Tissue-specific differentially methylated regions (T-DMRs) further regulate the DNA methylation landscape in adult brain cells, affecting the overall epigenetic profile (Hirabayashi et al., 2013). Given the diversity of cell types in the mammalian brain, certain genomic loci may exhibit bipolar DNA methylation pattern, meaning that these loci are hypermethylated in some cell subtypes and hypomethylated in others, reflecting the functional diversity across different cell populations (Sun et al., 2016). DNA methylation patterns exhibit significant variability across different brain regions and neuronal subtypes, and these epigenetic modifications are closely linked to the genetic risk of various neuropsychiatric disorders.

### 4.1 The role of DNA methylation across developmental stages

In brain development, DNA methylation plays a crucial regulatory role at various stages, particularly during postnatal neuronal maturation. During prenatal development, marked by early neural circuit formation, *de novo* methylation establishes tissue-specific patterns through DNMT1 (Costello, 2003). In the first trimester, the fetal brain undergoes global hypomethylation, especially in early stages of neural circuit formation. As development progresses into the second and third trimesters, DNA methylation levels gradually increase, particularly in gene body regions and non-promoter areas, shaping early neurodevelopmental pathways (Schneider et al., 2016).

Postnatally, methylation changes continue to influence synaptic plasticity and cognitive function, particularly throughout childhood, as neural networks are established to support higher-order cognition and memory consolidation (Schneider et al., 2016; Montesinos et al., 2018). During early childhood, characterized by rapid synapse formation, methylation is highly plastic during the first 5 years of life. Dynamic changes occurring at both CG and non-CG (CH) methylation sites, linked closely to neuron differentiation and function (Price et al., 2019), with over 7% of DNA methylation sites shifting in relation to gestational age and sex. These dynamic changes provide insight into early molecular mechanisms underlying neurodevelopmental disorders, such as schizophrenia and autism (Spiers et al., 2015).

As adolescence, a phase marked by synaptic pruning and increased neural complexity, DNA methylation continues to influence synaptic plasticity and cognitive functions. This restructuring phase includes critical epigenomic changes, particularly in the hippocampus and dorsolateral prefrontal cortex (DLPFC), establishing the foundation for neural circuit maturation (Price et al., 2019). In adulthood, as neural networks consolidate, DNA methylation patterns stabilize to support higher-order cognitive processes and memory consolidation. With age, global methylation gradually decreases (Jung and Pfeifer, 2015). Notably, in the prefrontal cortex (PFC), which matures later in development, DNA methylation changes most rapidly during fetal stages, slowing postnatally and decelerating further with aging (Numata et al., 2012). Methylation changes in aging are linked to neurodegenerative diseases, such as AD, where demethylation of neural function-regulating genes, such as *APP*, may lead to *β*-amyloid accumulation (Jung and Pfeifer, 2015).

### 4.2 DNA methylation patterns across brain regions

DNA methylation varies significantly across brain regions, contributing to the spatial regulation of cognitive function, emotional modulation, and neural transmission. The hippocampus, critical for memory and learning, exhibits lower methylation in active enhancers during early developmental stages (post-conceptional week 17; Jin et al., 2023). While some regions lose mCG marks as development advances, GABAergic neurons retain high hydroxymethylation (hmCG) levels in specific areas, highlighting unique regulatory needs during later development (Kozlenkov et al., 2018). In the DLPFC, which is essential for executive functions, both GABAergic interneurons and excitatory glutamatergic neurons (Glu) neurons undergo distinct methylation changes associated with genetic risk for neuropsychiatric conditions (Jin et al., 2023). The DLPFC also exhibits sex-biased methylation patterns in autosomal genes; for example, such as *GLUD1*, which encodes a key enzyme involved in glutamate metabolism and is associated with schizophrenia and cognition (Numata et al., 2012). In regions associated with emotion and reward, such as the nucleus accumbens (NAcc) and anterior cingulate cortex, hypomethylation in Glu and GABA neurons is closely associated with synaptic plasticity. Methylation changes in these regions play a pivotal role in the regulation of emotional behaviors, highlighting their importance in the formation of neural circuits responsible for emotional control and behavioral regulation (Rizzardi et al., 2019).

### 4.3 Cell type-specific methylation patterns

Aside from regional specificity, DNA methylation patterns also exhibit variability across distinct cell types. GABAergic and Glu neurons, for instance, exhibit distinct methylation landscapes: GABAergic neurons display a broader range of methylation changes, while Glu neurons show hypomethylation, especially in enhancer and gene body regions (Kozlenkov et al., 2018). Non-CpG methylation (mCpH) is widely present in both neuron types and is closely associated with gene expression, especially in regions distal to transcription start sites (Kozlenkov et al., 2016). Additionally, Glu neurons demonstrate significantly elevated levels of hmCG than GABA neurons, particularly in enhancer regions, indicating a role in active gene expression (Jin et al., 2023). Non-neuronal cell types, including microglia and oligodendrocytes (OLIG), demonstrate unique methylation profiles. Microglia, as resident immune cells in the brain, exhibit dynamically changing methylation patterns influenced by the neural environment. In cases of microglial depletion, infiltrating monocytes mimic certain aspects of microglial DNA methylation but retain epigenetic distinctions, underscoring the unique developmental trajectory of microglia (Lund et al., 2018). Oligodendrocytes regulate gene expression primarily through converting mCG to hmCG within gene bodies, with lower mCH levels exerting a lesser influence on expression (Kozlenkov et al., 2018).

### 4.4 Link between DNA methylation and neuropsychiatric disorders

Differential DNA methylation regulation across developmental stages, brain regions, and cell types not only governs brain development but also plays a critical role in neuropsychiatric disorder pathophysiology. Preterm birth, for instance, leads to substantial methylation alterations that disrupt white matter development, resulting in cognitive deficits and atypical neurodevelopment. DNA methylation patterns associated with gestational age suggest that epigenetic modifications could connect environmental factors, such as preterm birth, to long-term neurodevelopmental outcomes (Spiers et al., 2015; Wheater et al., 2022). Hypomethylated regions in Glu neurons overlap significantly with genetic risk loci for disorders such as schizophrenia, ASD, amyotrophic lateral sclerosis (ALS), and Parkinson’s disease, indicating that these epigenetic changes may be central to pathogenesis (Kozlenkov et al., 2018; Kozlenkov et al., 2016). Additionally, Purkinje neurons display hypomethylation in areas related to cerebellar development and activity, particularly at RORα binding sites, which are essential for the development of Purkinje neurons (Jin et al., 2023). Differentially methylated positions (DMPs) in the prefrontal cortex (BA10) are associated with neural development and higher-order processing during adolescence and memory consolidation in adulthood (Schneider et al., 2016).

Genetic mutations affecting DNA methylation highlight its crucial role in neuronal development and function. For instance, germline mutation in *DNMT3A* led to Tatton-Brown-Rahman syndrome (Yokoi et al., 2020), with some patients suffering from mild to severe intellectual disability. Similarly, hypomorphic mutation in *DNMT3B* was associated with immunodeficiency, centromere instability, facial anomalies syndrome, characterized by facial abnormalities and mental retardation (Jin et al., 2008). In mouse models, cKO of *Dnmt3b* in the dorsal CA1 region impaired hippocampus-dependent recognition memory (Kong et al., 2020). The inactivation of Dnmt3a and Dnmt3b during early embryonic development hindered *de novo* methylation (Feng et al., 2010). Conditional knockout of *Dnmt1* and *Dnmt3a* in forebrain excitatory neurons results in long-term plasticity abnormalities in the CA1 region, accompanied by learning and memory impairments (Feng et al., 2010). In neural stem cells, *Dnmt1* knockout disrupts dentate gyrus development and neurogenesis (Noguchi et al., 2016). In summary, these research findings collectively emphasize the foundational and complex role of DNA methylation mechanisms in regulating neuronal development, maintaining neuronal function, and supporting cognitive abilities.

## 5 DNA methylation in brain diseases

DNA methylation is of paramount importance for various biological functions, including disease onset (Ratovitski et al., 2022; Cholewa-Waclaw et al., 2019), aging (Sugden et al., 2022), memory (Lavery et al., 2020), and brain development. Recent studies increasingly reveal DNA methylation as a key regulator in several brain-related disorders, including SZ, ASD, ID, HD, and AD. These disorders display notable alterations in DNA methylation patterns across specific brain regions, including the prefrontal cortex, hippocampus, and temporal lobe in SZ, the hippocampus in AD (Altuna et al., 2019), and the striatum in HD (Pan et al., 2016). These methylation changes are closely associated with the dysregulated expression of genes involved in neural signaling, cell survival, and synaptic plasticity. Emerging evidence suggests that DNA methylation holds promise as an early diagnostic marker and biomarker for disease progression. Current studies are also actively exploring non-invasive methods, such as the analysis of DNA methylation patterns in peripheral blood, as potential diagnostic tools for these conditions (Ridler, 2018). This review will summarize current findings on DNA methylation alterations across these disorders and provide a comprehensive discussion on its role in disease onset and progression.

### 5.1 Schizophrenia

Schizophrenia is a complex disorder influenced by both genetic and environmental factors. Despite sharing identical genetic backgrounds, monozygotic twins often display significant phenotypic differences in schizophrenia and other complex disorders, suggesting that factors beyond genetic variation contribute to disease risk. DNA methylation, a critical gene–environment interaction mechanism, offers insights into the “missing heritability” and phenotypic variability observed in schizophrenia patients (Castellani et al., 2015).

Currently, antipsychotic drugs remain the primary treatment for schizophrenia, though approximately 50% of patients experience only partial symptom relief (Lohoff and Ferraro, 2010). Common genetic variants alone do not account for the variability in treatment response, positioning DNA methylation as a promising area for exploring the underlying pathophysiology and therapeutic outcomes of schizophrenia (van Os and Kapur, 2009).

With advancements in sequencing technologies, genome-wide methylation profiling is now feasible in both brain and peripheral tissues. Studies reveal that DNA methylation in the prefrontal cortex of schizophrenia patients undergoes significant changes from fetal to postnatal development, correlating with alterations in gene expression and schizophrenia risk loci (Kowalec et al., 2019). DMPs in key brain regions implicated in schizophrenia, such as the superior temporal gyrus, prefrontal cortex, and hippocampus, have been identified (Hannon et al., 2021; Hannon et al., 2016), closely correlating with clinical phenotypes, cognitive deficits, and symptom severity (McKinney et al., 2022). Variably methylated positions (VMPs) in genes involved in neuronal function, including *C17orf53*, *THAP1*, and *KCNQ4*, are particularly notable for their role in regulating the age of onset and cognitive impairments in schizophrenia (Li et al., 2021; Kiltschewskij et al., 2024a). Moreover, unique methylation patterns in the DLPFC suggest that DNA methylation is essential for gene expression regulation and neural circuit development (Lin et al., 2021).

DNA methylation and demethylation processes dynamically influence schizophrenia’s development and pathology (Jaffe et al., 2016). For instance, hypermethylation of genes such as brain-derived neurotrophic factor (*BDNF*) and *SOX10* in the prefrontal cortex and hippocampus leads to reduced gene expression, impairing learning, memory, and neurodevelopment (Çöpoğlu et al., 2016; Chen et al., 2015; Fu et al., 2020). Conversely, hypomethylation of CHGA and LINGO1 in the prefrontal cortex is linked to increased expression and abnormalities in neuroendocrine function, myelination, and neurodevelopment (Ueda et al., 2021).

**Figure 6:**
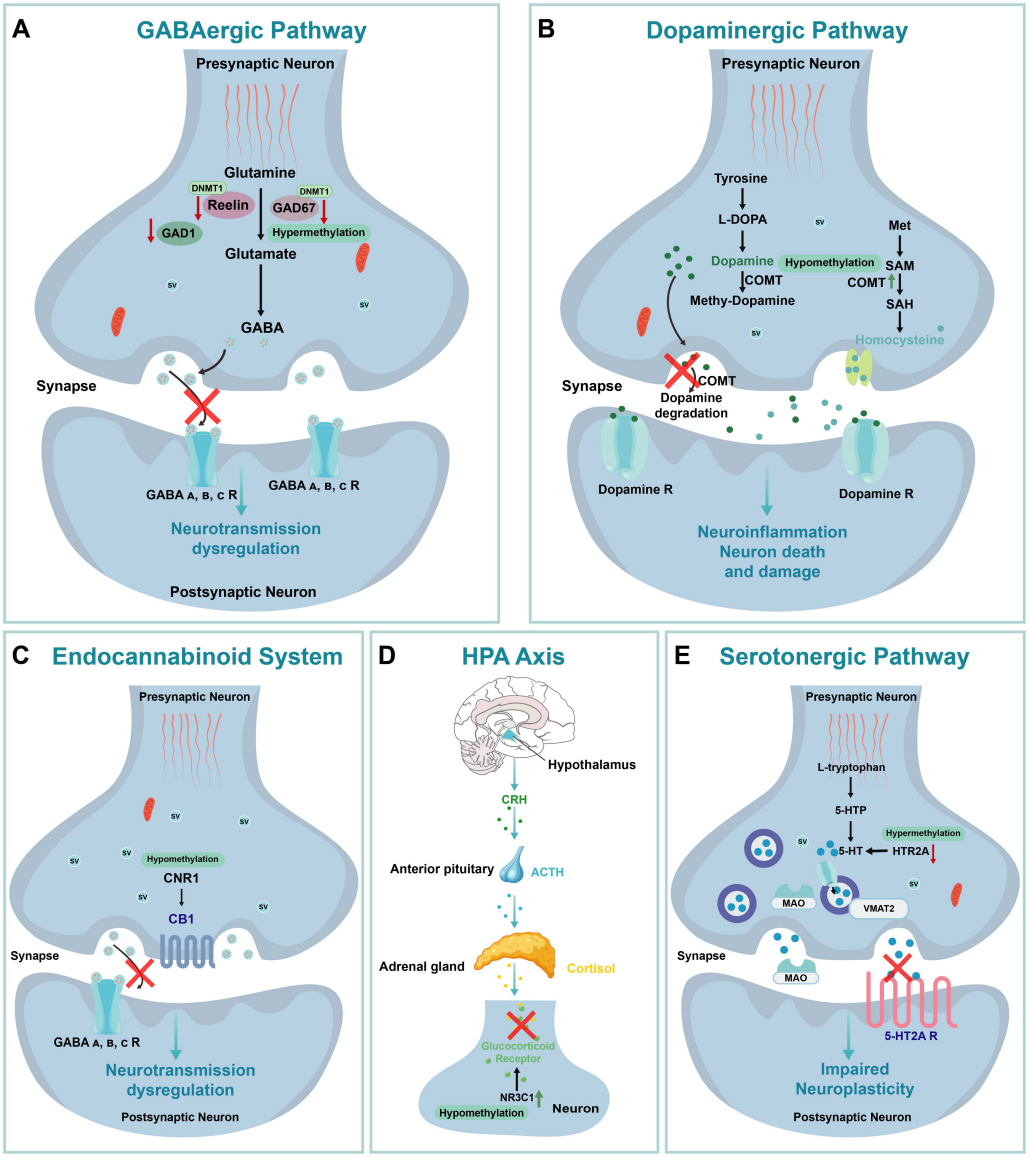
The Impact of DNA Methylation on Schizophrenia. **(A)** GABAergic pathway. GABA A, B, C R: Gamma-Aminobutyric Acid Type A, B, C Receptor. **(B)** Dopaminergic pathway. L-DOPA: L-3,4-dihydroxyphenylalanine; Met: Methionine; SAM: S-Adenosyl Methionine; SAH: S-Adenosyl Homocysteine. **(C)** Endocannabinoid system. CB1: Cannabinoid Receptor Type 1. **(D)** HPA Axis: Hypothalamic–Pituitary–Adrenal Axis; CRH: Corticotropin-Releasing Hormone; ACTH: Adrenocorticotropic Hormone. **(E)** Serotonergic pathway. 5-HTP: 5-Hydroxytryptophan; 5-HT: Serotonin; MAO: Monoamine Oxidase; VMAT2: Vesicular MonoAmine Transporter 2; SV: Synaptic Vesicle.

A substantial body of evidence highlights aberrant DNA methylation in schizophrenia at both site-specific and genome-wide levels, spanning multiple pathways implicated in the disorder. Within the GABAergic pathway, several key genes are dysregulated. Reelin (RELN), a glycoprotein primarily secreted by a subset of GABAergic interneurons, is essential cortical neural connectivity during prenatal development and for synaptic plasticity postnatally, both of which are crucial for cognitive function and disrupted in schizophrenia (Negrón-Oyarzo et al., 2016). DNMT1 overexpression in these neurons leads to DNA hypermethylation of *GAD67* and *RELN* promoters, resulting in decreased transcription, impaired synaptic function, and cognitive deficits (Grayson and Guidotti, 2013; Nabil Fikri et al., 2017; Figure 6A).

Elevated DNMT1 levels have also been observed in Brodmann Area 9 (BA9), a region crucial for cognitive functions commonly affected in schizophrenia (Tepest et al., 2008). In BA9, DNMT1 overexpression combined with *GAD67* hypermethylation disrupts GABAergic gene expression and neurotransmission. Pharmacological interventions, such as valproic acid and antipsychotic combinations, have shown partial success in reversing this methylation-induced dysregulation (Veldic et al., 2005). Additionally, methionine (MET) treatment, which raises S-adenosyl methionine (SAM) levels, a cofactor for DNMT1, further enhances promoter methylation and gene silencing in schizophrenia-related pathways (Grayson et al., 2009). Together, DNMT1 overexpression and the resulting gene silencing contribute to GABAergic dysfunction and neural circuit disruptions in schizophrenia. In this same region, hypermethylation of the glutamic acid decarboxylase 1 (*GAD1*) gene promoter, encoding the enzyme responsible for GABA synthesis, exacerbates deficits in GABAergic transmission in schizophrenia patients (Ruzicka et al., 2015; Figure 6A).

Similarly, dysregulation of DNA methylation in the dopaminergic pathway contributes to schizophrenia pathogenesis. Dopamine plays a critical role in cortical connectivity, and its dysregulation is associated with cortical dysfunction in schizophrenia (Horga et al., 2016). The catechol-O-methyltransferase (*COMT*), which is involved in dopamine metabolism, exhibits low promoter methylation correlated with increased gene expression, especially in male patients, suggesting an elevated schizophrenia risk (Gao et al., 2017; Figure 6B). In contrast, *DRD3* gene hypermethylation is associated with decreased expression, and dysregulated methylation of *COMT* and *DRD3* genes is linked to both schizophrenia risk and sex-specific differences (Dai et al., 2014). Notably, low *COMT* methylation has also been detected in saliva samples from schizophrenia and bipolar disorder patients, suggesting potential cross-tissue relevance and supporting the development of non-invasive biomarkers (Abdolmaleky et al., 2006). Since saliva is easier to collect than brain tissue, future research and biomarker development hold promise.

In addition, DNA methylation abnormalities extend to the endocannabinoid system (ECS), further implicating epigenetic regulation in schizophrenia. The promoter region of the cannabinoid receptor 1 (*CNR1*) gene, encoding the type 1 cannabinoid receptor, exhibited hypomethylation in the prefrontal cortex and peripheral blood mononuclear cells (PBMCs) of schizophrenia patients compared to control groups. This hypomethylation is consistent with elevated *CNR1* expression observed in MAM-induced animal models (Figure 6C; D'Addario et al., 2017), suggesting that *CNR1* methylation levels could serve as a potential biomarker for schizophrenia.

DNA methylation also plays a significant role in stress response mechanisms associated with childhood maltreatment (CM). CM has been shown to alter the hypothalamic–pituitary–adrenal (HPA) axis response to stress, leading to modified DNA methylation of the glucocorticoid receptor (*NR3C1*) and *BDNF* genes, which in turn increases susceptibility to schizophrenia (Barker et al., 2020; Figure 6D). Notably, *NR3C1* methylation exhibited sex differences, with significantly reduced methylation at the 1F-CpG32 site in female patients. This reduction may enhance the binding of the NGFI-A transcription factor, thereby promoting *NR3C1* expression and contributing to the onset and progression of the disease (Liu et al., 2020). Interestingly, previous studies have highlighted sex differences in the age of onset, negative symptoms, and affective symptoms in schizophrenia. While female patients demonstrated a higher burden of methylation dysregulation, they may possess certain biological mechanisms that mitigate more severe disease progression, potentially explaining the sex-specific differences in schizophrenia susceptibility (Zhou et al., 2023; Rukova et al., 2014).

DNA methylation within the serotonergic pathway is closely linked to schizophrenia phenotypes. The 5-HT2A receptor, encoded by *HTR2A*, is a critical regulator of fetal brain development and adult cognitive function (Paquette and Marsit, 2014). Previous studies have associated serotonin downregulation with schizophrenia (Shah and González-Maeso, 2019), and postmortem analyses of brain tissue from schizophrenia patients revealed significantly elevated methylation levels at the *HTR2A* promoter region (Cheah et al., 2017). In early-onset schizophrenia cases, methylation dysregulation of *MB-COMT* and *5-HT2A* was partially corrected by antipsychotic treatment, and hypermethylation of *5-HT2A* in the prefrontal cortex was correlated with treatment response (Abdolmaleky et al., 2006; Figure 6E). Additionally, low methylation levels of the *5-HT2A* gene were observed in the saliva of patients with schizophrenia and bipolar disorder, highlighting the potential of peripheral DNA methylation studies to identify novel biomarkers (Ghadirivasfi et al., 2011).

As previously discussed, DNA methylation changes in schizophrenia-related genes are integral to disease pathogenesis. Multiple studies have shown that specific antipsychotic medications can modulate DNA methylation levels in schizophrenia patients (Gillespie et al., 2024; Kiltschewskij et al., 2024b). For instance, clozapine and risperidone have been reported to alleviate symptoms in treatment-resistant schizophrenia by modulating the methylation of genes associated with peripheral white blood cells and cell adhesion (Kinoshita et al., 2017). In addition, increased methylation of the *DTNBP1* gene, observed in both schizophrenia patients and their first-degree relatives, was partially reversed with antipsychotic treatment (Abdolmaleky et al., 2015). Furthermore, elevated methylation of the *DTNBP1* gene in both schizophrenia patients and their first-degree relatives was partially reversed by antipsychotic treatments. In the prefrontal cortex, *5-HT2A* hypermethylation demonstrated a bidirectional regulatory effect, influencing treatment efficacy while also being modulated by medication (Abdolmaleky et al., 2006). This dual regulatory mechanism encompasses both stable and dynamic methylation patterns induced by treatment. Collectively, these findings underscore the potential of DNA methylation as a biomarker for schizophrenia and highlight its role in both the underlying biology of the disorder and patient response to treatment (Lokmer et al., 2023).

### 5.2 Autism spectrum disorders

Autism Spectrum Disorder is a complex neurodevelopmental disorder influenced by both genetic and environmental factors, with DNA methylation emerging as a significant contributor to its pathogenesis. Studies indicate that DNA methylation status during fetal development is closely linked to later neurodevelopmental abnormalities in ASD patients. Notably, DNA methylation changes in the placenta can influence neuron formation, synaptogenesis, and neural network connectivity, potentially disrupting genes essential for neurodevelopment and cell signaling (Bahado-Singh et al., 2021). Consequently, abnormal DNA methylation may lead to the dysregulation of ASD-related genes, which are crucial for brain function and development (Corley et al., 2019).

Genome-wide DNA methylation analyses support these early developmental changes, identifying differentially methylated genes and pathways in ASD, including *HLA-DRB5*, *FMN2*, and *CALCOCO2* (Sun et al., 2022), which may serve as early biomarkers for ASD diagnosis. Additional studies have pinpointed 58 DMRs in the prefrontal cortex of ASD patients, particularly associated with genes of the GABAergic system and brain-specific microRNAs (Nardone et al., 2017), suggesting that specific methylation changes in cortical neurons may underlie ASD-related neurodevelopmental abnormalities.

Among the genes implicated, *MECP2* stands out, given its association with Rett syndrome, a condition with clinical features overlapping with ASD. The MeCP2 protein regulates gene expression by binding to methylated DNA and interacting with histone deacetylase 1 (HDAC1), impacting neurodevelopment (Vuu et al., 2023; LaSalle and Yasui, 2009; Kinde et al., 2016). Although typically linked to Rett syndrome, *MECP2* mutations and reduced protein levels have also been observed in some ASD patients (Nagarajan et al., 2006), suggesting a potential pathogenic role in ASD. Supporting this, animal models reveal that *Mecp2* methylation in the hippocampus of mice reduces its expression, resulting in ASD-like behaviors (Lu et al., 2020). Similarly, in male ASD patients, increased methylation in the *MECP2* promoter region correlated with decreased expression (Samaco et al., 2005), and modest methylation increases in upstream boundary regions may facilitate methylation spread to the promoter. In contrast, *MECP2* methylation in female ASD patients deviates from typical X chromosome inactivation (XCI), indicating that methylation changes in females with ASD are likely locus-specific rather than affecting the entire X chromosome (Nagarajan et al., 2008). These sex-based differences provide insights into gender-specific methylation patterns in ASD.

The GABAergic system also plays a fundamental role in ASD neurobiology by maintaining excitatory-inhibitory balance in the brain; its dysregulation is implicated as a possible physiological basis for the disorder (Uzunova et al., 2016). The *GABRB3* gene, which encodes the β3 subunit of the GABA receptor, is essential for this balance. Mecp2 deficiency has been shown to reduce *GABRB3* expression in Mecp2-deficient mice and individuals with Rett syndrome and ASD, suggesting shared epigenetic pathways affecting *GABRB3* expression in these neurodevelopmental disorders (Samaco et al., 2005). Abnormal methylation of *GABRB3* may disrupt inhibitory neurotransmission, potentially contributing to the social and behavioral deficits in ASD (Zhang et al., 2019). Additionally, methylation of *GAD1* and *RELN* has been associated with neurodevelopmental abnormalities in ASD. Increased methylation in the *GAD1* promoter region enhances MeCP2 binding (Zhubi et al., 2014), leading to GAD1 repression in Purkinje neurons, potentially due to *in utero* epigenetic modifications that elevate ASD risk (Yip et al., 2007). Consistently, ASD mouse models show increased 5mC and 5hmC levels in the *GAD1* promoter, promoting MeCP2 binding and subsequent gene repression (Labouesse et al., 2015). Similarly, elevated methylation in the *RELN* promoter reduced *RELN* expression, particularly in the prefrontal cortex and cerebellum of ASD patients, potentially impairing neurodevelopment and impaired synaptic plasticity (Lammert and Howell, 2016; Zhubi et al., 2017). Thus, the epigenetic regulation of *GAD1* and *RELN* may significantly contribute to ASD-associated neurodevelopmental abnormalities.

While targeting DNA methylation in ASD treatment remains challenging, certain differentially methylated markers show promise as early diagnostic biomarkers. DMRs in the ASD placenta, enriched in loci related to synaptogenesis and neurogenesis, suggest that placental DNA methylation patterns could offer critical insights for early ASD prediction (Ravaei et al., 2023). Furthermore, non-genic DMRs reveal novel epigenetic mechanisms, presenting additional opportunities for clinical intervention in ASD. In summary, DNA methylation plays a multifaceted role in ASD development, with methylation abnormalities in genes such as *MECP2*, *GABRB3*, *GAD1*, and *RELN* indicating diverse pathological mechanisms, including neurodevelopmental disruption, neurotransmitter imbalance, and GABAergic system dysfunction. Future research should emphasize comprehensive methylation analyses across tissues and explore potential therapeutic modulation of DNA methylation, laying a scientific foundation for targeted interventions in ASD.

### 5.3 Intellectual disability

Intellectual disability is a prevalent neurodevelopmental disorder characterized by significant cognitive and adaptive functional impairments (Banerjee et al., 2022). Evidence suggests that specific alterations in DNA methylation play a central role in ID pathogenesis, affecting key neurodevelopmental processes such as neuronal maturation, synaptogenesis, and memory formation. Dysregulation in DNA methylation can lead to the aberrant expression of ID-associated genes, resulting in neurodevelopmental defects (Vasilyev et al., 2021). For instance, methylation abnormalities observed in brain tissue from certain ID patients are linked to atypical splicing of neurodevelopment-related genes, indicating that early epigenetic changes may critically influence later neurodevelopmental stages (Niggl et al., 2023).

There is considerable phenotypic and genetic overlap between ID and ASD, particularly in areas like cognitive impairments, social communication challenges, and behavioral difficulties, with both disorders sharing common epigenetic regulatory mechanisms. Mutations in key genes and disruptions in imprinted gene regulation are common to both ID and ASD. Notably, mutations in the *KCNK9* gene, located in the imprinted 8q24 region, have been associated with both ID (Birk-Barel syndrome) and certain cases of ASD, suggesting shared epigenetic pathways (Sánchez Delgado et al., 2014). Additionally, abnormal methylation of the *PEG13* gene has been proposed as a potential pathogenic mechanism common to both ID and ASD, underscoring the existence of overlapping gene regulatory pathways and highlighting DNA methylation’s regulatory role in these shared regions (Court et al., 2014). These shared mechanisms open the possibility for cross-condition therapeutic strategies that target gene methylation.

The *IMMP2L* gene on chromosome 7q31.1 has also been strongly linked to autism susceptibility (Viñas-Jornet et al., 2018). Microdeletions in this region result in reduced *IMMP2L* methylation, with partial compensatory effects observed in mothers of affected individuals, which may explain why some deletion carriers do not show clinical symptoms (Vasilyev et al., 2021). This phenomenon supports the concept of shared imprinted gene regulation between ID and ASD. Methylation patterns in imprinted genes such as *IMMP2L* suggest potential as biomarkers for predicting clinical variability, providing valuable insights for early diagnosis and personalized intervention.

Although ID and ASD share certain epigenetic characteristics, their regulatory pathways appear distinct from those in SZ. Genome-wide methylation analyses reveal that SZ and ID share methylation changes in genes like *CYP2E1*, *FYN*, *STAT3*, *RAC1*, and *NR4A2*, which are involved in neurodevelopment and immune function (Zhang et al., 2023). However, methylation dysregulation in SZ predominantly affects the dopaminergic and GABAergic systems, whereas in ID and ASD, it primarily influences neurodevelopmental genes. This divergence implies that, despite some overlap in gene regulation, SZ is characterized by unique epigenetic pathways influencing neurotransmitter systems. These findings illustrate how methylation dysregulation can lead to distinct neuropsychiatric outcomes, underscoring the importance of disorder-specific epigenetic approaches in treatment.

Additional evidence for gene-specific methylation in ID pathogenesis comes from studies on X-linked intellectual disability (XLID). Mutations in the *KDM5C* gene are a significant cause of XLID, accounting for approximately 3% of male cases (Wu et al., 2022). In one study involving monozygotic twins and their older brother, a frameshift mutation in *KDM5C* was identified, inherited from their asymptomatic mother, who exhibited skewed X-inactivation. DNA methylation analysis identified 399 differentially methylated CpG sites, mostly hypomethylated and concentrated in brain development-related regions (Guerra et al., 2020). This finding underscores *KDM5C*’s role in ID and suggests that gene-specific methylation patterns may directly influence ID phenotypes, highlighting the value of identifying methylation signatures to better understand genetic contributions to ID severity.

DNA methylation studies in peripheral blood samples from ID patients have also identified numerous methylation differences. Differential methylation in ID-associated genes (e.g., *COLEC11*, *SHANK2*, *GLI2*, and *KCNQ2*) and imprinted genes (e.g., *FAM50B* and *MEG3*) suggests that some ID patients may have undiagnosed imprinting disorders (Kolarova et al., 2015). These findings support the utility of DNA methylation analysis as a tool for detecting gene dysregulation across the course of ID, and suggest that methylation differences may contribute to the clinical heterogeneity observed among ID patients, further highlighting DNA methylation’s role in ID pathogenesis (Zhang et al., 2023). While current research has established associations between gene dysregulation and neurodevelopmental deficits mediated by DNA methylation, the precise regulatory mechanisms and their contributions to ID remain unclear. Future studies should focus on elucidating how early epigenetic changes affect ID progression and investigating their potential applications in personalized diagnostics and therapies.

### 5.4 Huntington’s disease

**Figure 7:**
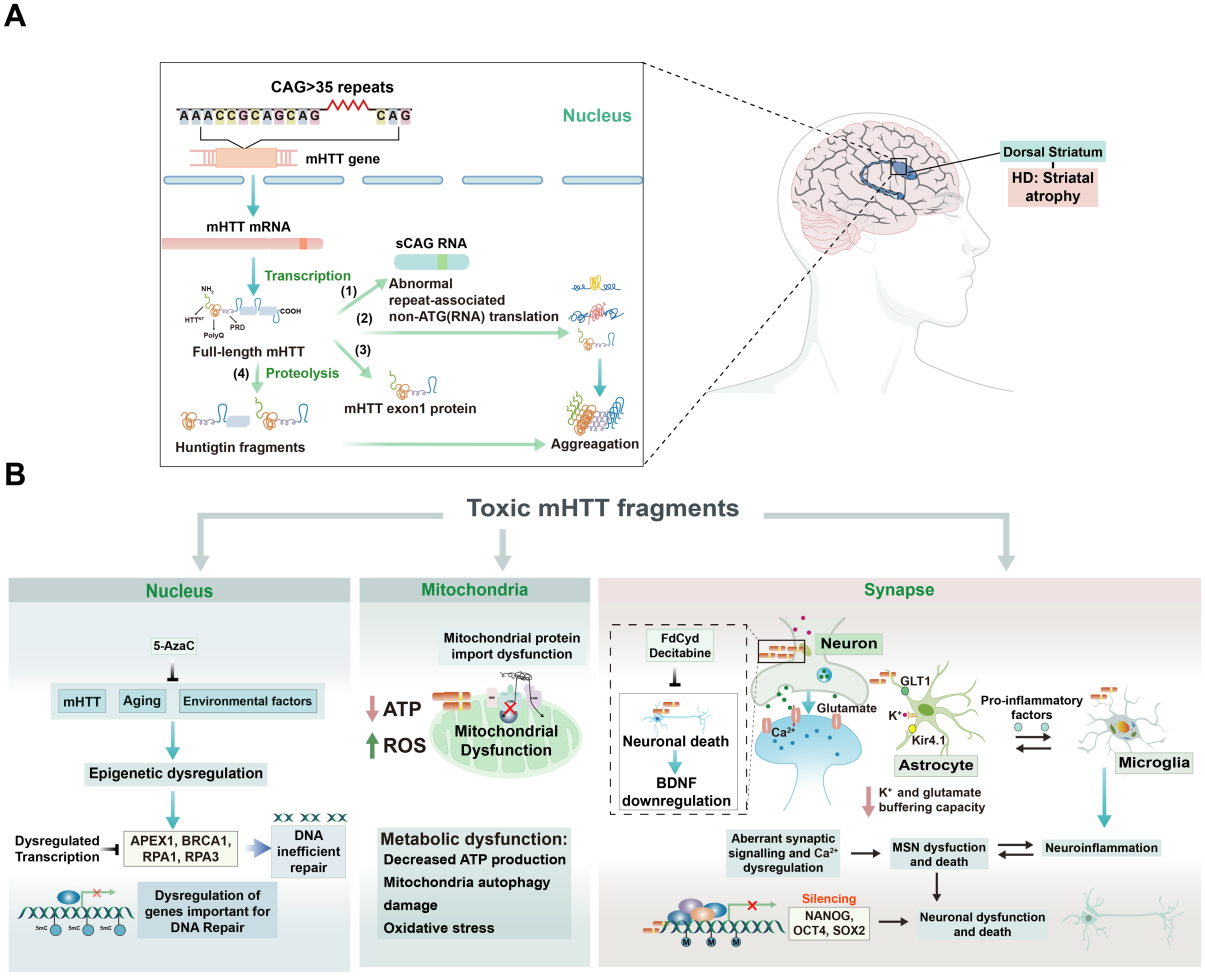
The role of DNA methylation changes in the pathogenesis of HD and the cellular impact of toxic huntingtin protein variants. **(A)** Production of toxic huntingtin protein variants. The huntingtin gene generates various toxic protein variants through multiple pathways, including RNA hairpins, abnormal repeat-associated non-ATG (RAN) translation protein products, amino-terminal huntingtin protein exon 1 protein fragment, and small fragments from full-length mutant huntingtin (mHTT). These fragments aggregate to form large inclusions in the cytoplasm and nucleus, contributing to cellular toxicity. **(B)** Cellular impact of toxic mHTT fragments. Nucleus: Toxic mHTT fragments disrupt nuclear processes, leading to transcriptional dysregulation and impaired DNA repair mechanisms. Factors such as DNA methylation changes, aging, and environmental influences exacerbate these effects, resulting in dysregulated transcription of important genes, including those involved in DNA repair. Mitochondria: mHTT fragments cause mitochondrial dysfunction, characterized by decreased ATP production, impaired mitochondrial protein import, autophagy damage, and increased oxidative stress, which contribute to metabolic dysfunction. Synapse: Toxic mHTT fragments affect synaptic function, leading to abnormal synaptic transmission, neuronal death, and decreased brain-derived neurotrophic factor (BDNF) levels. This results in altered synaptic plasticity, dysregulated neurotransmitter release, and neuronal dysfunction, ultimately progressing to cell death. Interventions with 5-AzaC, 5-Fluoro-2′-deoxycytidine (FdCyd), and decitabine have been shown to reverse transcriptional dysregulation and synaptic dysfunction, highlighting potential therapeutic strategies.

HD, an autosomal dominant genetic disorder, is primarily caused by the expansion of CAG trinucleotide repeats in the huntingtin (*HTT*) gene (Mollica et al., 2016; Kay et al., 2016; Figure 7A). The accumulation of toxic protein fragments disrupts various cellular functions, including mitochondrial protein import (Yano et al., 2014), synaptic integrity (Peng et al., 2018), and transcriptional regulation (Figure 7B).

Genome-wide cytosine methylation changes in an HD model were first observed in mouse striatal neurons expressing either pathogenic (STHdhQ111) or normal (STHdhQ7) polyQ domains. Reduced representation bisulfite sequencing (RRBS) revealed that genes exhibiting expression changes in low-CpG regions also demonstrated methylation alterations in the presence of mutant HTT (mHtt). Transcription factors such as AP-1, SOX2, FRA-2, and JUND were implicated in the regulation of cell differentiation, with *AP-1* binding sites overlapping those of FRA-2 and JUND, suggesting their collaborative role in gene regulation. In early HD models, key developmental genes, *Pax6* and *Nes* exhibited reduced binding to FRA-2 and JUND in the absence of Sox2. Increased methylation further diminished AP-1 binding in DMRs. Additionally, 4,808 regions fully methylated in wild-type cells displayed partial methylation in mutant cells, and 1,039 fully methylated regions in mHtt showed partial methylation in wild-type cells. In HD patient-derived cells, a downregulation of DNA repair genes (*APEX1*, *BRCA1*, *RPA1*, and *RPA3*), which are involved in mismatch and loop-out repair pathways was observed. Notably, *APEX1* was confirmed to exhibit promoter hypermethylation, and treatment with 5-AzaC restored normal expression of *APEX1* while inhibiting the expansion of the trinucleotide repeat (TNR) region (Mollica et al., 2016). These findings highlight the complex interplay between methylation changes and transcription factor binding in HD pathogenesis, suggesting potential targets for therapeutic intervention.

Mutant Htt appears to influence the epigenetic age of HD patients. Variations in CpG site methylation within the *Htt* gene have been observed in the mouse *Htt* gene knock-in model, the transgenic *HTT* sheep model, and humans (Lu et al., 2020). These variations are associated with alterations in epigenetic age, which depend on the clinical severity of the disease (Vonsattel et al., 1985). The length of CAG repeats is inversely correlated with the age of disease onset (Layburn et al., 2022), where longer repeats lead to earlier symptom manifestation (Farrer et al., 1992). This highlights the direct impact of CAG repeats on disease progression and suggests their potential role in accelerating epigenetic aging, which may contribute to the pathogenesis of HD by altering the biological aging process in tissues. Notably, DNA methylation alterations in the brains of HD patients have resulted in significant epigenetic age acceleration (AgeAccel) in regions such as the frontal lobe, parietal lobe, and cingulate gyrus, which has been linked to earlier onset of symptoms (Horvath et al., 2016). Consistent with this, the largest DNA methylation study of HD to date documented AgeAccel effects in the blood of HD cases (Lu et al., 2020), further indicating that DNA methylation changes play a crucial role in HD progression (Ng et al., 2013).

As previously expounded, disrupted DNA methylation has also been explored in HD mouse models. In the YAC128 mouse model, it was observed that levels of 5hmC in the striatum and cortex were significantly lower than in age-matched controls, with 5hmC primarily enriched in gene bodies, including intragenic CpG islands. These differentially hydroxymethylated regions (DhMRs) were enriched with genes associated with neurogenesis and neuronal survival (Kumar et al., 2018), involving pathways such as *Wnt*/*β*-*catenin*/*Sox*, *NMDAR*/*calcium*/*CREB*, and GABA type A receptor pathways. This suggests that dysregulated DNA methylation in these regions may serve as an important early contributor to HD pathogenesis. In the striatum, the global reduction in 5hmC may be attributed to the downregulation of *Tet1*, *Tet2*, and *Tet3*, along with the upregulation of *MeCP2*, while in the cortex, it may be related to the downregulation of *Tet1* (Wang et al., 2013).

The CpG methylation of certain genes implicated in the pathogenesis of HD has been analyzed. The adenosine A_2_A receptor (A_2A_R), a G-protein-coupled receptor essential for the survival of the basal ganglia, is highly expressed in the striatum, particularly in striatopallidal medium spiny neurons (MSNs) (Zhang et al., 2016). Downregulation of *ADORA2A* was associated with increased levels of 5mC and decreased levels of 5hmC in the 5′ untranslated region (UTR) region of *ADORA2A* in the putamen of HD patients. This finding is consistent with observations in R6/2 mice, where 5hmC levels were reduced at specific CpG sites within exon m2 of *ADORA2A*. This leads to speculation that changes in DNA methylation states may serve as potential biomarkers for the diagnosis of HD (Villar-Menéndez et al., 2013).

Global alterations in DNA methylation have revealed a link between the transcriptional dysregulation of *Bdnf* and its promoter methylation, with *Bdnf* being a key factor downregulated in HD patients and various HD models. In primary cortical neurons expressing *mHtt*, *mHtt* induced hypermethylation of the *Bdnf* promoter. Treatment with 5-Fluoro-2′-deoxycytidine (FdCyd) restored *Bdnf* expression in these neurons, alongside the expression of several neural-related genes, including *Drd2*, *Ppp1r1b*, *Penk*, *Rasd2*, and *Adora2a*, in R6/2 mice carrying the mutant human *HTT* gene (Pan et al., 2016). *Twist1*, which plays a crucial role during embryonic development and tumor transformation (Qin et al., 2012), exhibited increased expression in HD patient tissues and the R6/2 model. RNA interference (RNAi)-mediated knockdown of *Twist1* was shown to eliminate the *mHtt*-induced hypermethylation of the *Bdnf* promoter (Pan et al., 2018). Together, DNA methylation changes and the dysregulation of *Twist1* contribute to the downregulation of *Bdnf* in HD, underscoring the significance of epigenetic modifications in the pathogenesis of this disorder.

DNA methylation changes at specific loci hold promise as potential biomarkers for Huntington’s disease (HD), as such alterations are characteristic of the condition. Several genes have been identified as markers of disease progression. For instance, in blood samples from HD patients, hypomethylation of *PEX14*, *GRIK4*, and *COX4I2* has been linked to motor progression. *GRIK4* is particularly noteworthy, due to its selective expression in the cortex and striatum, where it plays a role in sensory motor gating and protection against excitotoxicity (Lu et al., 2020). Furthermore, using the Illumina Infinium Human Methylation27 Bead Chip microarray, significant differences in methylation levels of *CLDN16*, *DDC*, and *NXT2* were observed in HD patients compared to controls (Zadel et al., 2018).

Studies have shown that DNMT inhibitors can improve certain pathological features in HD models. However, it is crucial to recognize that manipulating epigenetic mechanisms can have both positive and negative effects. While some interventions may reduce trinucleotide repeat instability and heighten *Bdnf* levels, they could also increase the instability of *LINE-1* elements and lead to HTT hypomethylation, which is associated with disease progression. There, a more comprehensive understanding of HD pathogenesis is essential to accurately assess the risks and benefits of targeting DNA methylation in therapeutic approaches.

### 5.5 Alzheimer’s disease

**Figure 8:**
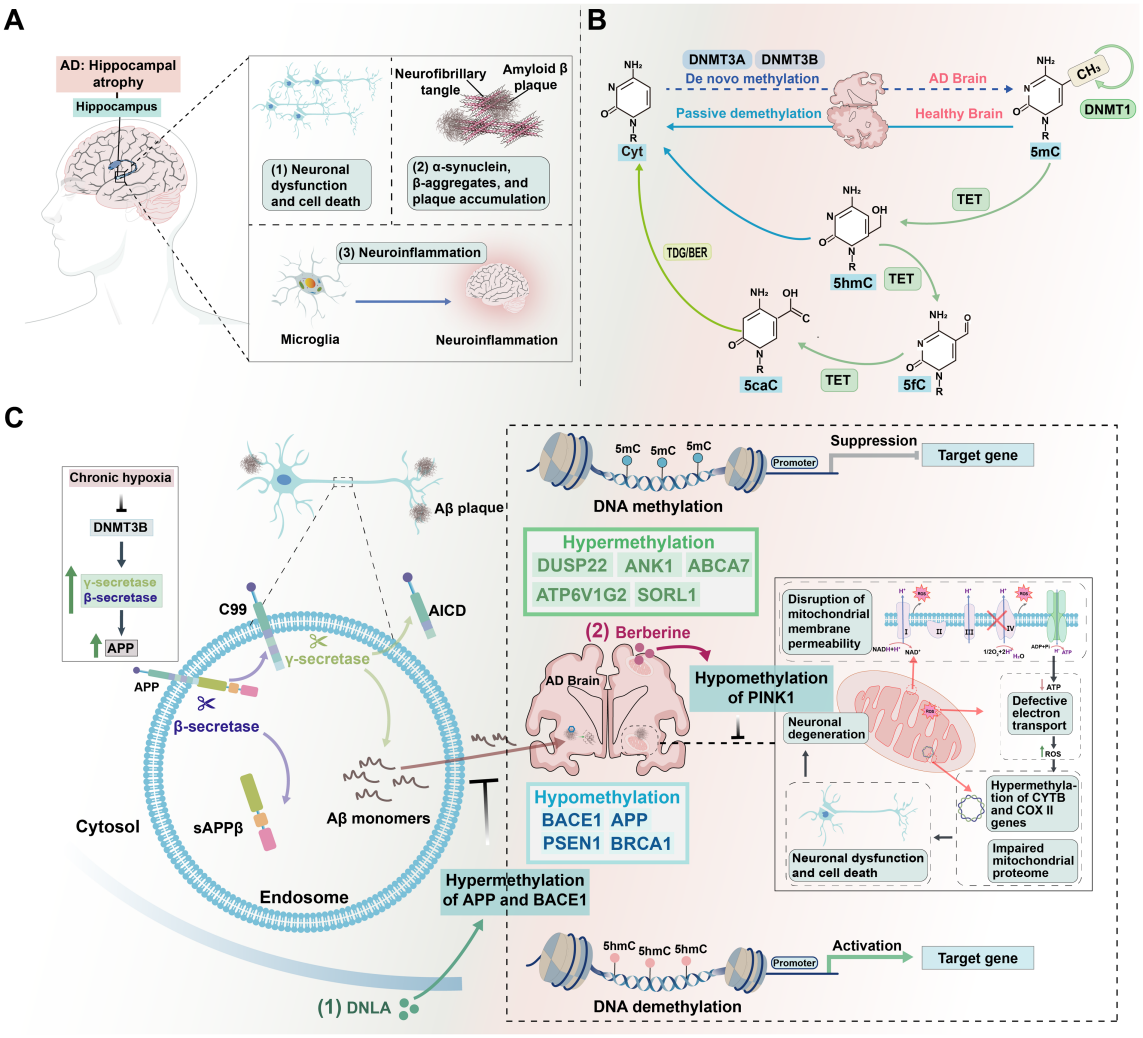
The critical roles of DNA methylation in AD. **(A)** Impact of AD pathology on hippocampal structure. AD pathology in the hippocampus is characterized by neuronal dysfunction and degeneration, activation of inflammatory responses, and abnormal deposition of Aβ plaques and tau protein neurofibrillary tangles. These factors collectively contribute to the progression of AD. **(B)** DNA methylation differences between AD and healthy brains. In healthy brains, DNMT1 maintains DNA methylation, while DNMT3A and DNMT3B facilitate *de novo* methylation. TET enzymes convert 5mC to 5hmC, 5fC, and 5caC, which are then demethylated back to cytosine. In AD brains, this balance is disrupted, leading to abnormal methylation patterns that contribute to disease progression. **(C)** Influence of DNA methylation changes on AD progression. In AD, DNA methylation balance is disrupted, resulting in hypermethylation and hypomethylation of specific genes, altering their expression. Hypoxic conditions exacerbate this imbalance by inhibiting DNMT3B expression, triggering increased deposition of Aβ and tau proteins, and worsening AD pathology. Mitochondrial dysfunction, including disrupted membrane permeability and impaired electron transport chain activity, leads to oxidative stress. This is accompanied by abnormal hypermethylation of mitochondrial genes such as *CYTB* and *COX II*, along with decreased mitochondrial DNA (mtDNA) copy numbers, resulting in neuronal death and accelerating AD progression. (1) Alkaloids (DNLA) reduces Aβ production by promoting the hypermethylation of *APP* and *BACE1*; (2) Berberine maintains mitochondrial homeostasis by promoting the hypermethylation of *PINK1*. Additionally, MRZ-99030 mitigates Aβ deposition by targeting and neutralizing Aβ oligomers.

AD is a progressively deteriorating neurodegenerative disorder characterized by cognitive decline and neuronal death (Tang et al., 2023). Autosomal dominant AD is associated with specific mutations in the amyloid precursor protein (*APP*), presenilin 1 (*PSEN1*), or *PSEN2* genes, while late-onset AD is significantly influenced by the presence of two copies of apolipoprotein ɛ4 allele (Fetahu et al., 2019). Aging is the primary risk factor for late-onset AD and is associated with chronic inflammation in the CNS. In the aging brain, the accumulation of Aβ and mitochondrial dysfunction contribute to the sustained activation of microglia, thereby maintaining a chronic inflammatory state (Bajwa et al., 2019; Andronie-Cioara et al., 2023). In AD brain tissue, a decrease in SAM led to DNA hypomethylation (Morrison et al., 1996), which in turn upregulated genes associated with the formation of neuritic plaques such as *CACNA1H*, *PSEN1*, and PEN2 (Zhao et al., 2017; Liu et al., 2016). Studies have also found that reduced levels of 5mC and 5hmC in the hippocampus were negatively correlated with increased amyloid plaque pathology (Chouliaras et al., 2013; Figure 8B), similar to age-related changes in 5mC and 5hmC observed in the APP^swe^/PS1^ΔΕ9^ mouse models of AD (Chouliaras et al., 2018). These findings indicate that alterations in DNA methylation may critically influence the onset and progression of AD by modulating signaling pathways of Aβ accumulation.

Specifically, DNA hypomethylation accelerated the progression of Aβ and tau pathways. Aβ interacted with multiple receptors, activating signaling pathways that promoted neuroinflammation and neurodegeneration (Heneka et al., 2015; Figure 8A). This process subsequently led to synaptic dysfunction, neuronal loss, memory impairment, and cognitive decline (Morris et al., 2014). For instance, Promoter hypomethylation activated *CASPASE-4*, which boosted *IL-1β* release, intensifying neuroinflammation and accelerating Aβ plaque formation (Krause et al., 2019). The activation of *CASPASE-4*, due to promoter hypomethylation, enhanced the release of IL-1β, aggravating neuroinflammation and accelerating Aβ plaque formation (Daily et al., 2024). Similarly, DNA demethylation following Aβ-induced DNA damage led to the upregulation of *BRCA1*, a key DNA repair gene, in AD mouse models (Tarsounas and Sung, 2020). However, in a tau-dependent manner, BRCA1 was mislocalized to the cytoplasm, resulting in DNA fragmentation and further accelerating the progression of AD pathology (Mano et al., 2017).

The cleavage of APP by beta-site amyloid precursor protein cleaving enzyme 1 (BACE1) generated a soluble sAPPβ ectodomain and a 99-amino-acid C-terminal membrane-bound fragment. Subsequent cleavage of this fragment by *γ*-secretase produced Aβ40 and Aβ42 peptides, both of which were central to the aggregation of neuritic plaques, and BACE1 activity was recognized as an early biomarker of AD (Nicsanu et al., 2022). Hypomethylation of enhancers in the *DSCAML1* gene in AD neurons led to the activation of *BACE1*, resulting in the overproduction of Aβ peptides and the eventual formation of amyloid plaques. In turn, Aβ peptides induced enhancer hypomethylation in genes regulating neurogenesis and cell cycle, driving the formation and propagation of tangle pathology, ultimately leading to neuronal death and the cognitive deficits characteristic of AD (Li et al., 2019). An alternative therapeutic strategy for AD could involve inhibiting *BACE1* overactivation by preventing the hypomethylation of *DSCAML1* or *CASPASE-4*. This would target the pathological triggers of *BACE1* overexpression and might consequently minimize the side effects. Targeting the epigenetic dysregulation responsible for *BACE1* overexpression may mitigate the pathological drivers of Aβ plaque formation while minimizing associated side effects.

Moreover, the hypermethylation of genes such as *ELOVL2*, *ATP6V1G2*, *DUSP22*, *ANK1*, *ABCA7*, and *SORL1* was implicated in the regulation of Aβ and tau pathways (Kim et al., 2023; Sanchez-Mut et al., 2014; Furuya et al., 2012; de Jager et al., 2014; Yu et al., 2015; Blanco-Luquin et al., 2020). Among these, the *ELOVL2* gene played a pivotal role in cellular processes, including membrane stabilization and inflammation, both of which directly influenced Aβ and tau dynamics. Notably, increased methylation levels of *ELOVL2* were positively correlated with p-tau protein deposition in the hippocampus. Moreover, alterations in *ELOVL2* methylation were associated with changes in fatty acid synthesis and immune response regulation, both of which were integral to AD pathology (Blanco-Luquin et al., 2020). This suggests that inhibiting the DNA methylation of *ELOVL2* could represent a potential therapeutic strategy for AD.

Mitochondria provide the energy required for synaptic transmission, synaptogenesis, and synaptic pruning via oxidative phosphorylation (OXPHOS). Disruptions in mitochondrial structure and function can induce oxidative stress, with the resulting ROS causing damage to mtDNA, thereby accelerating neurodegeneration (Klemmensen et al., 2024). Mitochondrial dysfunction is a key factor in AD pathogenesis, as mitochondria are responsible for regulating both cellular metabolism and apoptosis (Tian et al., 2023; Figure 8C). The expression of genes within mtDNA was also regulated by DNA methylation. In most AD samples, mtDNA methylation in the D-loop region is decreased (Stoccoro et al., 2017). Conversely, elevated mtDNA methylation has been observed in the *CYTB* and *COX II* genes, accompanied by reduced expression of the mitochondrial gene 12S rRNA (Xu et al., 2019; Xu et al., 2021). D-loop demethylation may compensate for the hypermethylation of genes encoding 12S rRNA, *CYTB*, and *COX II*. The *PINK1* (PTEN-induced kinase 1)-Parkin (Parkin RBR E3 ubiquitin protein ligase) pathway, which is essential for mitochondrial quality control, played a key role in maintaining mitochondrial homeostasis. Berberine, a mitochondrial-targeting compound, can inhibit *PINK1* promoter hypermethylation and enhance *PINK1* expression, thereby promoting autophagy-lysosome pathway and alleviating mitochondrial dysfunction in AD models (Wang et al., 2023).

Efforts have been made to identify specific DNA methylation markers for AD to assess risk factors, track disease progression, and identify biomarkers. A key element is the methylation status of repetitive *LINE-1* elements, which significantly contribute to global DNA methylation patterns. In blood samples from AD patients, elevated *LINE-1* methylation levels were observed as a response to DNA damage that enhances the affinity of DNMTs for these regions. This suppressed the activation of transposable elements and prevented genomic instability (Bollati et al., 2011). Studies have also shown that during AD and aging, *LINE-1* transcriptional activation has increased in the brain, particularly in the context of tau pathology-driven chromatin relaxation (Guo et al., 2018). Activated LINE elements generate abundant RNA–DNA hybrids, which are recognized as foreign nucleic acid signals by the cGAS-STING pathway, inducing immune responses and amplifying inflammatory signals closely linked to neuroinflammation in AD. Inhibition of c-Jun can prevent LINE activation and the accumulation of RNA–DNA hybrids, thereby reducing cGAS-STING pathway activation and alleviating neuronal programmed cell death and inflammation (Guo et al., 2018; Scopa et al., 2023). This reactivation of *LINE-1* may lead to genomic instability, thereby promoting neurodegenerative processes. Type I interferon (IFN-I) is a key regulatory factor in immune responses, and the high expression of *LINE-1* is closely associated with its production. When *LINE-1* is overexpressed, immune cells may mistakenly recognize *LINE-1* transcripts as foreign nucleic acids, triggering an innate immune response and promoting the production of IFN-I. Typically, the *LINE-1* promoter region is inhibited by high methylation levels, suppressing its expression. However, in certain diseases, low methylation of the *LINE-1* promoter leads to its overexpression, which in turn activates immune factors such as IFN-I, resulting in immune dysregulation and inflammatory responses (Mavragani et al., 2016). Consequently, *LINE* hypermethylation in immune cells may act as a compensatory mechanism to suppress LINE-1 activity and maintain genomic stability (Scopa et al., 2023). These findings highlight the tissue-specific regulation of LINE: hypermethylation in the blood may serve as a predictive biomarker for AD, while increased LINE expression in the brain may directly contribute to disease progression. Immune cells, unlike neurons, do not undergo the same degree of chromatin relaxation, thereby retaining the repressive effects of DNA methylation, indicating a protective role in genomic stability.

Additionally, hypomethylation of the *BIN1* gene at specific CpGs (26, 44, and 87) in the blood has been associated with an increased risk of LOAD (Salcedo-Tacuma et al., 2019), while hypermethylation of the *PIN1* gene promoter distinguished frontotemporal dementia (FTD) from the hypomethylation observed in AD (Ferri et al., 2016). These findings demonstrate that DNA methylation plays a significant role in the progression of AD, potentially even before clinical symptoms appear. As DNA methylation sequencing technologies advance, more specific biomarkers for AD diagnosis are likely to be identified. Certain treatments targeting methylation demonstrate neuroprotective effects. In a mouse model of AD induced by a high methionine diet (HMD), alkaloids (DNLA) increased methylation levels of *APP* and *BACE1*, reduced DNMT1, and upregulated DNMT3a and DNMT3b, thereby alleviating AD-like symptoms (Pi et al., 2022). This strategic targeting of DNMTs in regulating Aβ accumulation offers a novel therapeutic approach that holds promise for innovative treatments addressing the challenges posed by AD.

## 6 Concluding remarks

In recent years, research on DNA methylation has expanded from oncology to the neurological field, encompassing both the brain and the retina. DNA methylation plays a pivotal role in various biological functions, including the development of the retina and brain, as well as in their associated diseases (Lu et al., 2023). Dysregulation of DNA methylation has been linked to neurological disorders, affecting genomic stability and gene expression (Horvath and Raj, 2018). During retinal development, DNMTs regulate the differentiation of retinal progenitor cells into photoreceptor cells, with DNMT deficiencies leading to impaired retinal morphology and severe visual deficits (Singh et al., 2017). Similarly, in brain development, DNMTs regulate neuronal maturation and maintenance by modulating gene expression at key loci (Tognini et al., 2015).

DNA methylation plays a multifaceted role in neurodevelopment, influencing brain maturation and retinal homeostasis, and is critically involved in the progression of neurological disorders such as schizophrenia, ASD, ID, AD, and HD. Notably, SZ, ASD, and ID display distinct DNA methylation alterations across various brain regions, reflecting unique pathophysiological mechanisms. In SZ, aberrant DNA methylation patterns in the prefrontal cortex and hippocampus are associated with disruptions in synaptic plasticity, neurotransmitter signaling, and neuronal development, contributing to cognitive and behavioral dysfunction. Similarly, in ASD and ID, DNA methylation changes affect key genes involved in neuronal connectivity, neurogenesis, and synapse formation, essential for brain development and function. These DNA methylation alterations are linked not only to neurodevelopmental deficits but also to enduring impacts on cognitive abilities and behavior, highlighting their significance in the etiology and progression of these disorders.

Given the focus on retinal diseases such as DR, glaucoma, and AMD has been growing, understanding the role of DNA methylation in both retinal and brain diseases is becoming increasingly important. The visual signals received by the retina are transmitted to the brain via the optic nerve, a concept that closely links retinal diseases with brain diseases, such as glaucoma and AD (Erskine and Herrera, 2014). Understanding the role of DNA methylation in these contexts may help uncover the potential connections between the retina and the brain. AD patients often experience visual impairments that precede other dementia-related symptoms (Moons and De Groef, 2022; Shi et al., 2021; Yoon et al., 2019), and these visual changes are associated with the loss of RGCs and the degenerative processes (Asanad et al., 2019). Interestingly, Aβ deposition is observed in both AMD and glaucoma, reflecting pathological features similar to those in AD (Parsons et al., 2015). In the retina, Aβ aggregation not only affects neurotransmission and cellular signal transduction but may also trigger microglial activation, influencing signaling pathways between the retina to the brain that are modulated by DNA methylation (Donato et al., 2023). Reducing Aβ production by upregulating DNA methylation at the *BACE1* promoter could represent a shared mechanism across these tissues (Huang et al., 2018; Pi et al., 2022). Additionally, hypermethylation of the *LINE-1* promoter is observed in both AMD and AD, potentially reflecting the influence of environmental factors on DNA methylation in these diseases. The compound MRZ-99030 has demonstrated neuroprotective effects in animal models of glaucoma and AD by inhibiting Aβ aggregation, further supporting the potential link between brain and retinal diseases (Parsons et al., 2015).

While these findings underscore the potential of DNA methylation as a therapeutic target, it is essential to recognize that simple modifications in DNA methylation may not yield comprehensive solutions due to the complex and dynamic nature of this epigenetic process. The development of DNA methylation-based “epigenetic clocks” that reflect aging, when combined with disease-specific DNA methylation alterations, holds promise for identifying biomarkers and predicting disease risk (Horvath and Raj, 2018; Salameh et al., 2020). This review highlights key DNA methylation changes across various genes and their association with different diseases (Table 2), with a focus on genes that exhibit altered methylation patterns that may serve as biomarkers for diagnosis or disease progression.

**Table 2:** Potential risk DNA methylation changes associated with mechanisms of neurodegenerative diseases and their treatment strategies.

Disease	Predominant cell types affected	Susceptible genes	Effects of DNA methylation on mechanism	Pathological Characteristics	Treatment strategies	Refs
AMD	Photoreceptor and retinal pigment epithelium	GSTM1, GSTM5	Hypermethylation of the GSTM1 and GSTM5 promoters contributed to AMD by reducing their protein expression and increasing retinal sensitivity to oxidative stress	Irreversible central blindness Choriocapillaris loss in areas underlying intact retinal pigment epithelium cells Vascular dropout; Decreased vascular density	Intravitreal aflibercept injections Laser therapy Anti-VEGF therapies: ranibizumab, aflibercept, bevacizumab, brolucizumab Emerging therapies: Targeting the complement system	Hunter et al. (2012)
						Sohn et al. (2019) and Fleckenstein et al. (2021)
DR	Photoreceptor and retinal vascular endothelial	MMP9	MMP9 hypomethylation led to mitochondrial damage and accelerated apoptosis in capillary cells	Visual impairment and blindness Microaneurysms and intraretinal microvascular abnormalities, capillary blockage and retinal ischemia	Systemic therapy: Lipid-lowering therapy Multifactorial intervention Ocular therapy: Surgical intervention Anti-VEGF therapies Focal laser coagulation Intraocular steroids	Mohammad and Kowluru (2020)
		ECM1	Low methylation of ECM1 promoted retinal neovascularization			Cai et al. (2024)
Glaucoma	Retinal ganglion cell GABAergic neurons	HSP70	Hypermethylation of the HSP70 gene is correlated with decreased HSP70 protein expression, which may weaken the cellular stress response and thereby promote the development of glaucoma in patients with pseudoexfoliation syndrome	Loss of the neuroretinal rim and widening of the cup in the optic disk Optic nerve head loss of the neuroretinal rim Enlargement of the optic cup Deepening of the optic cup Thinning of the retinal nerve fiber layer and optic disk hemorrhages	Open-angle glaucoma: Laser therapy Surgical intervention Drug Treatment: (e.g., latanoprost, tafluprost) Chronic open-angle glaucoma: Drug Treatment: (e.g., timolol, latanoprost, brimonidine) Primary angle-closure glaucoma: Peripheral laser iridotomy Drug Treatment: (e.g., timolol, latanoprost, brimonidine, pilocarpine) Congenital glaucoma: Surgical intervention: (e.g., goniotomy or trabeculotomy)	Jonas et al. (2017)
						Hayat et al. (2020)
		GDF7	TET enzymes maintained hypomethylation of the GDF7 promoter, leading to trabecular meshwork fibrosis and aqueous humor outflow obstruction in primary open-angle glaucoma			Wan et al. (2021)
SZ	GABAergic neuron	GAD67	Increased GAD67 promoter methylation reduces its expression, decreasing inhibitory neurotransmission and impacting excitatory neuron activity	Positive Symptom Negative Symptom Cognitive impairment Disorganization syndrome	Dopamine receptor-blocking drugs Psychological intervention Cognitive behavioral therapy	Grayson and Guidotti (2013) and Jauhar et al. (2022)
HD	Striatal and cortical neuron	HTT	Abnormal DNA methylation in mutant huntingtin-induced neurotoxicity downregulated DNA repair pathway genes (APEX1, BRCA1, RPA1, and RPA3) and contributed to cell death	Involuntary choreic movements, behavioral changes, and cognitive impairment Degeneration of neurons in the striatum and the deeper layers of the cerebral cortex	Drug Treatment: (e.g., tetrabenazine, deutetrabenazine)	Mollica et al. (2016)
AD	Hippocampal and cortical neuron	APP, PSEN1, PSEN2	Abnormal DNA methylation of the APP, PSEN1, and PSEN2 genes disrupted their expression, leading to disturbances in the APP metabolic pathway. This disruption contributed to the formation of amyloid plaques and resulted in neurotoxicity	Memory impairment, aphasia, apraxia, agnosia, visuospatial skills impairment, executive dysfunction, and personality and behavioral changes Cognitive impairment	Drug Treatment: Aducanumab, Lecanemab, Donanemab AChEI: tacrine, donepezil, rivastigmine, galantamine NMDA receptor antagonists: memantine	Fetahu et al. (2019)

DNA methylation plays a fundamental role in neurodevelopment, influencing brain maturation, retinal health, and the progression of both neurodegenerative and neurodevelopmental disorders, including AD, HD, SZ, ASD, and ID. Each condition displays distinct DNA methylation patterns, yet overlapping mechanisms related to neurotransmitter regulation, synaptic plasticity, and cognitive function reflect the broader implications of DNA methylation in both neurodevelopmental and neurodegenerative processes.

In AD, DNA methylation changes not only mirror neurodegenerative pathways but also involve early neurodevelopmental processes such as neurogenesis and synaptic formation. Similarly, HD shows methylation changes affecting neurodevelopmental genes, while SZ displays dynamic methylation patterns across multiple neurodevelopmental stages. These shared and distinct methylation mechanisms suggest that investigating DNA methylation’s role in these diseases could provide an integrative perspective on its effects across the brain and retina.

A more nuanced understanding of DNA methylation in health and disease will expand our knowledge of epigenetics, especially in relation to diseases of the retina and brain. However, genomic studies using post-mortem or disease-affected tissues present inherent challenges, potentially obscuring cell-specific DNA methylation patterns. To overcome these challenges, future research should focus on single-cell analyses and investigate the interactions between DNA methylation and other epigenetic modifications. Additionally, cross-disciplinary collaboration between ophthalmology and neurology researchers and clinicians is crucial, as it will deepen our understanding of the connections between retinal and brain diseases and, ultimately, improve patient outcomes.
